# Statistical methods for measuring trends in colorectal cancer incidence in registries: A systematic review

**DOI:** 10.3389/fonc.2022.1049486

**Published:** 2022-11-30

**Authors:** Norah Alsadhan, Alaa Almaiman, Mar Pujades-Rodriguez, Cathy Brennan, Farag Shuweihdi, Sultana A. Alhurishi, Robert M. West

**Affiliations:** ^1^ Department of Community Health Sciences, College of Applied Medical Sciences, King Saud University, Riyadh, Saudi Arabia; ^2^ Leeds Institute of Health Sciences, School of Medicine, University of Leeds, Leeds, United Kingdom

**Keywords:** incidence, trends, colorectal cancer, methods, population-based study, systematic review

## Abstract

**Background:**

Monitoring cancer trends in a population is essential for tracking the disease’s burden, allocating resources, and informing public health policies. This review describes variations in commonly employed methods to estimate colorectal cancer (CRC) incidence trends.

**Methods:**

We performed a systematic literature search in four databases to identify population-based studies reporting CRC incidence trends, published between January 2010 and May 2020. We extracted and described data on methods to estimate trends and assess model validity, and the software used.

**Results:**

This review included 145 articles based on studies conducted in five continents. The majority (93%) presented visual summaries of trends combined with absolute, relative, or annual change estimates. Fourteen (10%) articles exclusively calculated the relative change in incidence over a given time interval, presented as the percentage of change in rates. Joinpoint regression analysis was the most commonly used method for assessing incidence trends (*n*= 65, 45%), providing estimates of the annual percentage change (APC) in rates. Nineteen (13%) studies performed Poisson regression and 18 (12%) linear regression analysis. Age-period-cohort modeling- a type of generalized linear models- was conducted in 18 (12%) studies. Thirty-nine (37%) of the studies modeling incidence trends (*n=*104, 72%) indicated the method used to evaluate model fitness. The joinpoint program (52%) was the statistical software most commonly used.

**Conclusion:**

This review identified variation in the calculation of CRC incidence trends and inadequate reporting of model fit statistics. Our findings highlight the need for increasing clarity and transparency in reporting methods to facilitate interpretation, reproduction, and comparison with findings from previous studies.

## Introduction

Quantifying and monitoring cancer incidence in a population are essential for tracking the disease burden and resource planning. Observing changes in cancer rates over time can enhance our understanding of its historical evolution, the potential social and environmental risk factors leading to cancer, and the impact of implementing interventions and policies. To produce reliable findings on population-level incidence trends, investigators usually rely on population-based cancer registries for providing valid cancer data.

Recent years have witnessed an extensive focus on studying the epidemiology of colorectal cancer (CRC). CRC is a major global health problem, and its incidence rate has increased over the past decades. According to the GLOBOCAN 2020 estimates of cancer incidence, CRC is the third most common cancer and the second leading cause of cancer-related deaths worldwide (1). In CRC, survival outcomes are associated with the clinical stage at diagnosis (2); thus, it is one of the few cancers where screening is considered a critical preventive measure (3). Many population-based reports and epidemiological studies have investigated trends in CRC incidence over time and what societal, environmental, or political changes have been related to these transitions in incidence. Time trend analysis of CRC by age group has also been critical in developing and evaluating secondary prevention efforts such as screening programs (4, 5).

Different methods have been utilized to assess CRC incidence trends. Visual summaries in the form of graphs and descriptive tables are widely used, most often complementing the use of advanced statistical methods. A well-known, established approach for quantifying trends is the estimated annual percentage change (APC), representing the yearly average change in incidence rate. The APC is usually estimated by computing the regression model’s slope fitted to the log-transformed incidence rates (6). Different statistical models have been used to estimate the APC, such as linear, Poisson, and joinpoint regression. Some modeling strategies account for age, calendar period, and birth cohort effects on incidence trend estimates (7). The derived inferences from these modeling techniques are imperative for directing resource allocation and public health policies. Yet, the integrity of these inferences largely depends on the modeling procedure’s validity. Thus, previous methodological studies have underscored the importance of assessing model validity, including evaluating candidate models, selecting the final model, and assessing model assumptions or performance (8, 9).

When choosing the statistical software to conduct the trend analysis, it is essential to note that different software uses different methods and permits different outputs to be reported. Also, not all software requires the same technical skills; some need coding experience, while others are considered user-friendly in terms of learning the tool and implementing the analysis. Therefore, researchers should be aware of the most commonly used tools to assess trends and what output is usually reported.

To our knowledge, no previous study has examined and summarized the methods used to assess incidence trends in the literature and the extent of reporting model validity assessment. This review was set up to answer the following questions: 1- What are the various statistical methods reported in the literature for assessing CRC incidence trends, and what type of parameters are reported? 2- What model validity measures are reported in studies using statistical modeling? 3- What software is employed to conduct the analysis?

The current study was conducted in parallel with a comprehensive review describing incidence rate measures and evaluating the quality of reporting incidence methods (10).

## Methods

The reporting of this systematic review followed the Preferred Reporting Items for Systematic Reviews and Meta-Analyses (PRISMA) (11).

### Study identification

In May 2020, we searched Embase, Medline, Web of Science, and the Cochrane Library for studies reporting temporal trends in CRC incidence, published since 2010. In consultation with an information specialist, we developed a search strategy that included keywords and a combination of subject headings, including “colorectal cancer,” “incidence,” “trends,” and “registry” (the complete search strategy is provided in **Supplementary File 1**. We also checked the reference lists of identified studies to detect potentially missed articles.

### Study selection

We included studies that fulfilled all of the following criteria: 1) population-based retrospective studies using registry data to measure and report the incidence trends of colorectal cancer, 2) written in English, and 3) a full text is published. We excluded studies conducted in selected population groups (i.e., CRC incidence trends amongst patients with specific diseases), measured the incidence of multiple cancer types, or only reported trend estimates calculated in previous research. Furthermore, studies published as commentaries, case studies, clinical trials, case-control studies, reviews, conference proceedings, abstracts, or posters were excluded from this review.

### Selection process

**Figure 1:**
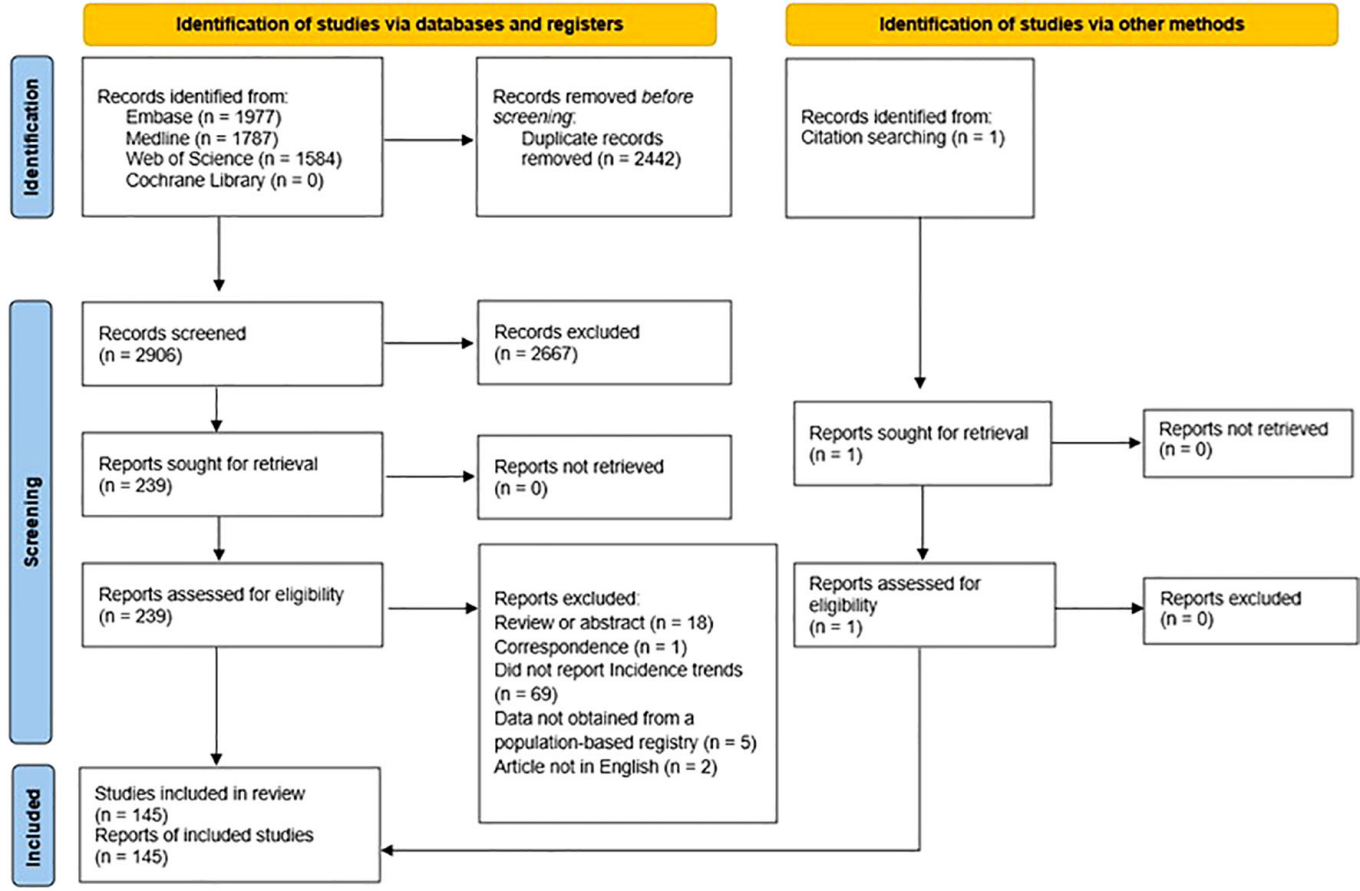
PRISMA flowchart of the study selection process.

**Figure 1** summarizes the selection process. After importing all potential abstracts into the screening web app “Rayyan” (12), two independent reviewers screened all titles and abstracts against the inclusion-exclusion criteria to exclude clearly irrelevant articles. Reviewers resolved disagreements through discussion, and in cases where a consensus decision was not reached by screening the title and abstract, the reviewers examined the full text. We used Cohen’s *κ* statistic to calculate the inter-reviewer agreement rate for title/abstract screening. After the screening process, we further assessed all articles selected for full-text review. If no consensus was reached, we consulted a third reviewer.

### Data extraction and synthesis

One reviewer independently extracted the data using a standardized form pilot-tested in ten studies. The form included details on the author and publication year, country, main study outcomes, observation period, methods for calculating incidence trends, model fit statistics (if applicable), and the software used. To ensure the robustness of the extraction process, a second reviewer cross-checked a random sample of 25% (*n=*36). Discrepancies were resolved by consensus agreement.

### Quality assessment

We assessed the quality of all included studies using a prespecified checklist adapted for this review and based on the Joanna Briggs Institute Critical Appraisal tool for prevalence studies (13) and the Appraisal tool for Cross-Sectional Studies (14). We chose relevant criteria from each tool to create a 10-item checklist for this study (**Supplementary Table 2.1** **)**. Items were assigned a score of 1 if “demonstrated in the study” or 0 if “not demonstrated or unclear”. We calculated and presented an overall score for each study, with higher scores indicating studies of higher quality. This overall quality score should be interpreted cautiously, as each quality indicator’s scores are often subjectively justified (15).

### Data analysis

The general characteristics of included studies and the methods utilized to assess incidence trends were described using descriptive statistics, reported as frequencies and percentages.

### Methods used to measure incidence trends

**Figure 2:**
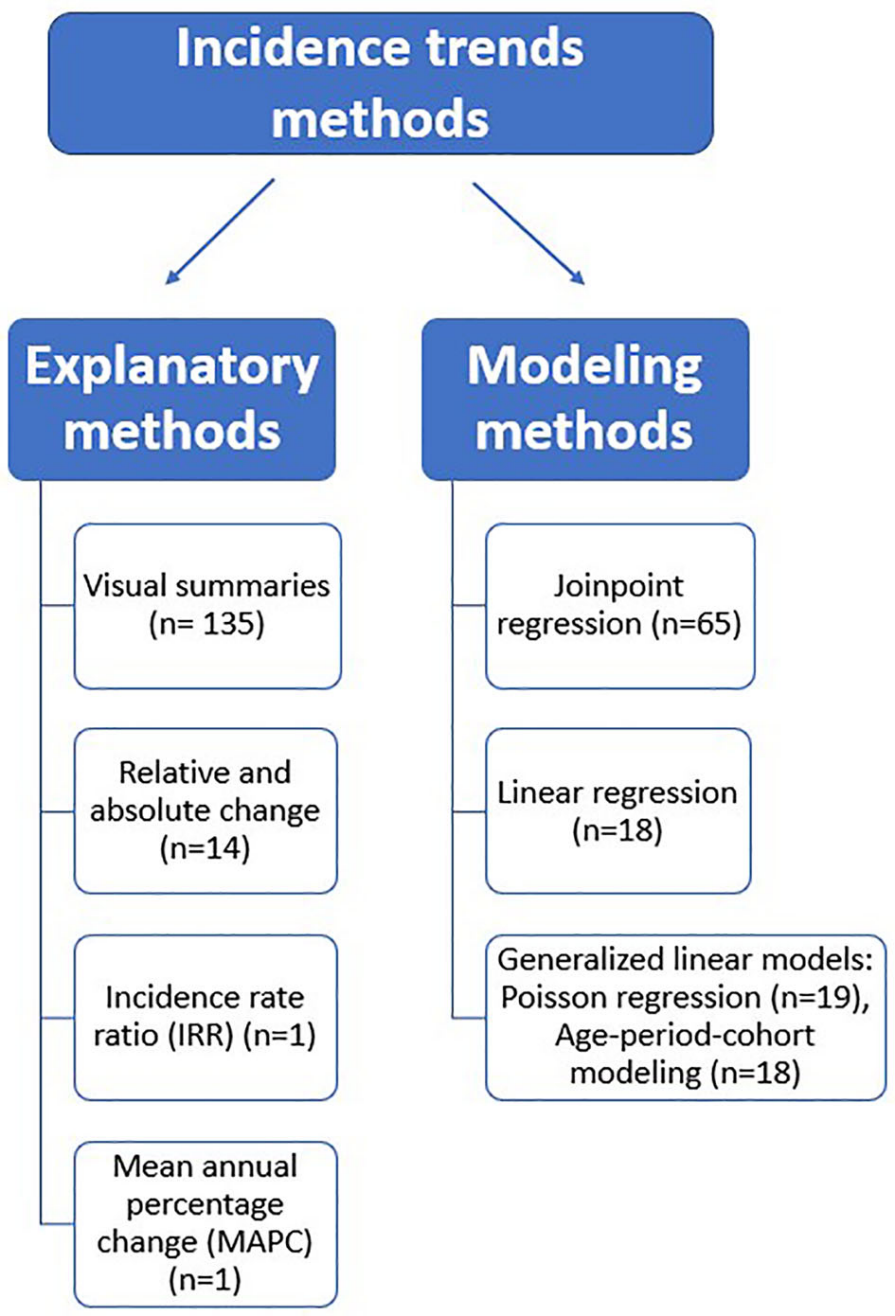
Classification of incidence trends methods.

Based on the findings of this review, we classified the reported methods into explanatory and modeling methods (see **Figure 2**).

#### Explanatory methods

Our definition of explanatory methods included visual summaries (graphical and tabular presentation of trends) and simple arithmetic calculations employed to estimate incidence trends.

#### Modeling methods

The reviewed modeling methods used regression analysis to fit a relationship between a dependent variable (incidence rate) and an independent variable (time). This review’s most commonly reported statistical modeling methods were subdivided into the following groups: joinpoint regression, linear regression, and generalized linear models such as Poisson regression and age-period-cohort models. The choice of the modeling method will depend on the researcher’s aim. When the interest is only to examine the annual percentage change in rates, linear or Poisson regression would be an appropriate option. However, joinpoint regression would be a suitable choice when the research aims to identify points in time where rates change in direction (join points) and magnitude. Thus, joinpoint analysis is especially useful when evaluating trends over a long time period when diagnostic techniques have changed, or relevant prevention or screening interventions have been implemented. Additionally, studies focusing on disentangling the simultaneous and independent effect of age (biological processes of aging), birth cohorts (exposures/experiences that vary from one generation to the next), and period (external factors that affect all age groups similarly at a specific calendar time) on cancer incidence should use age-period cohort modeling methods. **Supplementary File 2** provides a summarized description of each of the aforementioned statistical techniques.

## Results

The combined search initially yielded 5,348 articles, and after removing duplicates, we identified and screened 2,906 titles. Of these, 2,667 studies were excluded at the title/abstract level, and another 94 were excluded after full-text assessment. The remaining 145 articles were included in the review. The inter-reviewer agreement for the title/abstract screening had a Cohen’s *κ* value of 94% (**Supplementary Table 1**). The PRISMA flow diagram (**Figure 1**) shows details of excluded reports.

### Characteristics of included studies

The eligible articles comprised studies from North America, including the United States of America (*n*=58, 40%) and Canada (*n*=3, 2%), Europe (*n*=34, 23%), Asia (*n*=33, 23%), Oceania (*n*=6, 4%), Africa (*n*=3, 2%), and eight (6%) multi-country study. In addition to incidence, the two other study outcomes most commonly reported were mortality (*n=*35, 24%) and survival (*n=*30, 21%). Overall, 21 (14%) studies covered a study period of fewer than ten years, and the remaining covered ten years or more of observation. The characteristics and details of the studies (16–160) are provided in **Supplementary Tables 3, 4**. Detailed quality score information for all included studies is presented in **Supplementary Table 2.2**.

### Summaries of incidence trends methods

#### Explanatory methods

The majority of included studies (93%) presented trends using visual summaries combined with other statistical methods. Yet, 23 (16%) studies analyzed trends through only graphical and tabular presentations of incidence rates. Garcia et al. (88) and Murphy et al. (134) reported the absolute change in incidence, representing the arithmetic difference between any two rates at different time points. Fourteen articles assessed trends by calculating the relative change in incidence over a given time interval, presented as the percentage of change in rates. None of these studies reported confidence interval estimates, and only two indicated the significance of trends (75, 140). Cheng et al. (39) and Garcia et al. (82) provided details on the calculation employed for deriving the relative change; the following formula was reported:


$$Relative\;change=\frac{\begin{array}{c} Final\;rate-Initial\\ \;rate\;(absolute\;change) \end{array}}{Initial\;rate}\times 100$$

In one study, Nooyi et al. (87) measured trends by calculating the mean annual percentage change using the formulae:


$$\text{Mean annual percentage change}=\left[\frac{Final\;rate-Initial\;rate}{\left(Initial\;rate\times number\;of\;years\;between\;Final\;and\;initial\;rate\right)}\right]\times 100$$

Furthermore, Fedewa et al. (85) used incidence rate ratios to illustrate changing patterns in incidence by comparing more recent years versus earlier ones.

#### Statistical modeling methods

##### Joinpoint regression

Joinpoint regression analysis (*n*= 65, 45%) was the most commonly used method for assessing incidence trends by fitting a series of joined straight lines to estimate annual percentage change (APC) in incidence rates. More than half (*n=* 37, 57%) of the studies using this method calculated and reported only the APC. Of these, 15 (40%) reported one APC for the entire observation period, whereas 22 (59%) reported several APCs for different time segments during the study period. Eleven (17%) studies only reported the calculation of the average annual percentage change (AAPC), and 16 (25%) studies reported both the APC and AAPC. Only four studies explicitly stated the difference in calculation between the APC and AAPC. Further details on the estimated trend parameters and the number of joinpoints in the regression model are provided in **Supplementary Table 5**. The joinpoint trend analysis software developed by the National Cancer Institute (NCI) (161) was the only tool reported for this type of regression modeling. Examining the reporting of parameter setting in the joinpoint software revealed an overall inadequacy, with the model selection method being the most reported information (52%). Details on parameter setting reporting for the joinpoint program are provided in **Supplementary Tables 4** and **5**.

##### Linear regression modeling

Of all included studies, 18 (12%) used linear regression analysis to estimate linear trends. The majority of them (89%) reported the percentage of change in rates. Chittleborough et al. (41) reported trends as the difference per decade, and Baniasadi et al. (26) reported only the model formulae with no estimates for trends. Only nine studies indicated the method used to fit the linear regression model. The least-squares estimation method was the only reported one, and eight studies further indicated that the weighted-least squares technique was employed. None of these studies justified chosen model estimation procedure. Five studies performed a log transformation of the linear model (23, 25, 59, 105, 111). Different software was used for linear regression analysis, with SPSS (162) being the most commonly used (*n=4*, 22%).

##### Generalized linear models

Overall, 19 (13%) employed a Poisson regression model to quantify changes in incidence rates. Sixteen of these studies reported measures such as the incidence rate ratio or percentage of change to illustrate incidence trends. Abdifard et al. (17) and Abdifard et al. (16) indicated incidence trends by merely presenting the slope of the regression line. In one study, Dehghani et al. (46) explained only the significance of the incidence trend with no reporting of any other parameter indicating the pattern of rates over time. Five studies reported the use of Poisson regression to conduct age-period-cohort analysis. Only two studies (28, 63) reported consideration for dispersion, and only one (63) indicated the use of negative binomial distribution to correct overdispersion. The most commonly reported software for conducting Poisson regression was Stata (163) (*n=*6, 31%), followed by SAS (164) (*n=*5, 26%).

Age-period-cohort modeling- another type of generalized linear models - was employed to measure incidence trends in 18 (12%) studies. The model’s parameters used to estimate trends varied across these studies. The period/cohort rate ratio (ratio of rates in a specific period/cohort relative to reference period/cohort) was the most reported measure presented in 14 studies. Although reference values are usually arbitrarily chosen, nine studies in this review used the middle calendar period and birth cohort as reference categories. Chambers et al. (34) and Wessler et al. (149) took the earliest periods and cohorts as the reference, while Siegel et al. (114) chose the cohort with the lowest incidence rates. Estimations for the Local drift (age-specific net annual percentage change) and Net drift (age-adjusted annual percentage change) were indicated in seven and six studies, respectively. **Supplementary Table 4** provides details of all reported parameters. The most common software for this type of modeling was the publicly available age-period-cohort analysis web tool (*n=*8, 44%), developed by the NCI (165).

##### Other methods

Models that were reported only once in the reviewed literature included: time-series analysis (144), interrupted time-series analysis (83), bayesian analysis of spatio-temporal conditional autoregressive models (142), and the LOESS method (66). Six studies reported the APC without explaining the used model to derive this estimate (31, 35, 39, 121, 145, 159).

##### Association between number of years covered and the statistical method chosen

We further examined the studies to identify if the number of years studied had influenced the statistical method chosen (i.e., studies covering fewer years tend to use specific methods). We found no evidence to support any connection.

##### Model validity measures

Thirty-nine (37%) of the studies modeling incidence trends (*n=*104, 72%) explicitly indicated the method used to evaluate model fitness. Of all studies that utilized joinpoint regression analysis, 34 reported the method employed to select the final model. The permutation test (166), the only technique used, was either explicitly indicated in the text or cited in the reference list. Of these 34 studies, only five clearly reported the number of joinpoints in the best-fitting model.

Additionally, six studies (21, 34, 40, 62, 114, 149) indicated other approaches to assess model fitness. Of these, one study (21) failed to report the assessment’s result, while the remaining indicated a good model fit. Detailed information on the employed model fit statistics is provided in **Supplementary Table 4**.

### Software

Most studies (*n=*111, 77%) reported the software used for incidence trend analysis. The most common (52%) was the joinpoint program (161). Other reported software included SPSS (13%) (162), STATA (12%) (163), and SAS (11%) (164) (see **Supplementary Table 4**).

## Discussion

To our knowledge, this is the first study to examine variations in the methods employed in calculating incidence trends of CRC. The 145 articles retrieved provided valuable information on the most commonly reported methods and parameters for measuring trends.

### Methods used to measure incidence trends

#### Explanatory methods

Some studies in this review relied solely on explanatory methods to investigate trends. The exclusive use of visual summaries (graphical and tabular presentation of incidence) may lead to an erroneous and subjective interpretation of findings. We also noted that many studies that used only visual summaries covered an observation period of ten years or more. Yet, it was unclear why other methods were not used in conjunction with visual summaries.

Fourteen studies in this review reported trends as the relative change or percentage of change (PC) in rates between different periods. A positive PC corresponds to an increase in incidence rates, while a negative PC corresponds to a decreasing trend. Relative changes in incidence can be misleading because an absolute small difference can result in a significant percentage change. Therefore, it is important to provide readers with estimates of absolute and relative differences with confidence intervals to interpret incidence trends accurately.

#### Statistical modeling methods

This review identified different statistical modeling techniques for characterizing CRC incidence trends. The most commonly reported method was the joinpoint regression analysis using the NCI’s joinpoint program (161). Several APCs for varying periods could be generated depending on the number of joinpoints included in the model and the final selected model. Reporting the APC for each joinpoint segment provides a detailed description of how disease risk changes over time. Yet, to facilitate comparisons of incidence trends for various groups, it is essential to develop a summary measure of incidence trends that accounts for varying trends over sub-time intervals. Hence, in 2009, Clegg et al. (167) proposed the average annual percentage change (AAPC) as a summary measure of trends, computed as a weighted average of the slope coefficients of the joinpoint regression line, with weights corresponding to the length of each subinterval. The APC and AAPC have different interpretations, and thus, it is emphasized that both should be reported, if possible, to provide a comprehensive analysis of trends. The calculation of the AAPC has been incorporated into the joinpoint trend analysis Software (161).

In this review, 15 studies reported only one APC over the entire study period. These studies did not clarify why only a single APC was estimated, whether segmented analysis was not possible -due to the number of data points included- or has yielded insignificant findings, or if this measure reflects the AAPC. Eleven articles reported the AAPC without indicating if the final selected model provided APCs for different time segments, which would have provided an enhanced description of trends. Most studies failed to explain the interpretation of the AAPC, what it represents, and the calculation differences between the APC and AAPC. Providing the reader with a clear description of these parameters and their meaning is vital for understanding trends, reproducing findings, and making potential comparisons in future studies. Furthermore, this review highlighted inadequate reporting on the parameters set in the joinpoint program. Such details are essential for replicating the analysis or justifying when the researcher’s findings differ from previously published trends estimates using the same data source.

Poisson regression was the second most reported method in this review. Our results indicated underreporting of the verification of model assumptions concerning dispersion. Authors should inform readers if any model assumptions did not hold and how it was handled in the analysis.

When exploring cancer burden, disentangling the effects of age, cohort, and period is vital for a comprehensive analysis and understanding of incidence trends. Due to issues related to data availability and concerns about the statistical interpretability of age, period, and cohort analysis, researchers’ uptake and interest in this type of assessment were limited. To facilitate the conduction of this analysis, Rosenberg et al. (165) developed a freely available and easy-to-use web tool that provides researchers with a panel of estimable functions for age-period-cohort analysis. This tool was the most used in this review. Furthermore, we noted that nine studies used age-period-cohort modeling and joinpoint regression to analyze their data. This analysis approach of combining methods is imperative for strengthening the analysis, revealing emerging cancer trends, and enhancing our understanding of cancer etiology and natural history.

Among all studies that assessed and reported CRC incidence trends *via* statistical modeling, less than half reported model fit statistics in this review. Most of them focused on documenting the method used without further explanation of the model fit analysis. Examining model fitness is one aspect of assessing the statistical model’s validity; it is defined as “a measure of the discrepancy between the observed empirical distribution of the observations in the data set and the ‘best-fitting’ probability distribution computed from the estimated probability model” (8). Model fit statistics might include graphical assessment such as residual plots or quantitative evaluation such as log-likelihood tests and goodness-of-fit measures (8, 9). Despite the used methods, authors should provide details on model fit statistics in the manuscript or as supplemental or web-based data. Ensuring transparency by providing sufficient information on the modeling building procedure will support an accurate interpretation of the research findings and facilitate future analysis replication.

To our knowledge, this study is the first to review the methods employed to estimate incidence trends across different populations and settings. In cancer studies, the quality and reliability of the cancer registry data are essential for evaluating cancer trends. It was not within the scope of the current review to examine data quality reporting; yet, in a previous publication, we assessed reporting quality in CRC incidence studies and noted a substantial deficit in reporting registry-data quality control procedures and findings (10).

This review was limited to studies assessing the incidence of CRC using registry data. Thus, we might have missed other trend analysis methods used to analyze different data sources and diseases in the last decade. Despite this, our results inform well about a variety of commonly used incidence trends methods and thus support future researchers in choosing potential methods and parameters that will enhance the comparability of their research. Although we searched multiple databases and included studies from different countries, we included only English articles in this review. Thus, we might have missed relevant papers in other languages.

## Conclusion

This review described the most commonly reported methods for measuring CRC incidence trends over the past decade. Visual summaries are always a good starting point for observing trends, preferably followed by modeling. Joinpoint regression was the most reported method, identifying points in time where incidence rates change. We also noted an increased uptake of age-period-cohort modeling to disentangle the effect of age, period, and birth cohort on incidence trends. Our findings highlighted the need for increased clarity and transparency in reporting incidence trends methods to facilitate interpretation and comparison of results with previous studies and help identify and address limitations of the analysis.

## Data availability statement

The original contributions presented in the study are included in the article/**Supplementary Material**. Further inquiries can be directed to the corresponding author.

## Author contributions

NA, MP-R, RW, and FS conceived the idea for this review and the research questions. NA developed and conducted the search, screened the studies, performed data extraction and analysis, and wrote the first draft. AA participated in the screening process. SA participated in data extraction. RW, CB, and FS provided overall supervision and critically appraised the results. All authors contributed to the article and approved the submitted version.

## Funding

This study is part of Norah Alsadhan’s PhD study, funded by King Saud University, Riyadh, Kingdom of Saudi Arabia.

## Acknowledgments

The authors wish to acknowledge Natalie King (Information Specialist, University of Leeds) for her help with the development of the search strategy for this systematic review.

## Conflict of interest

The authors declare that the research was conducted in the absence of any commercial or financial relationships that could be construed as a potential conflict of interest.

## Publisher’s note

All claims expressed in this article are solely those of the authors and do not necessarily represent those of their affiliated organizations, or those of the publisher, the editors and the reviewers. Any product that may be evaluated in this article, or claim that may be made by its manufacturer, is not guaranteed or endorsed by the publisher.

## Abbreviations

APC, Annual percentage change; AAPC, Average annual percentage change; CRC, Colorectal cancer; NCI, National Cancer Institute; PRISMA, Preferred Reporting Items for Systematic Reviews and Meta-Analyses; PC, Percentage of change; SAS, Statistical Analysis System; SPSS Statistical Package for The Social Sciences.
