# Supplementary material for: Statistical methods for measuring trends in colorectal cancer incidence in registries: A systematic review
Source: Front Oncol. 2022 Nov 30;12:1049486. doi: 10.3389/fonc.2022.1049486 (PMC9748480; doi:10.3389/fonc.2022.1049486)
Supplement: Supplementary file 1 [file DataSheet_1.zip › Supplementary File 2.DOCX]

**Supplementary File 2 (**p.1-3**)**

**Descriptive summary of statistical modeling methods commonly used to measure incidence trends in 2010-2020**

**Modeling method**

***Joinpoint regression***

In evaluating incidence trends, join point (segmented) regression analysis identifies the points in time where rates change in direction (join points) and the magnitude of the incidence rate change. It estimates the percentage change using a permutation test-the most common technique reported in cancer incidence- to fit a series of joined straight lines to the natural logarithm of observed rates (1). The join point regression analysis starts with the minimum number of joinpoints (0 join points, which is a straight line) and tests whether more joinpoints are statistically significant and should be added to the model. When the join point model is fixed on zero joinpoints, the conventional annual percentage change (cAPC) is estimated. The cAPC assumes that incidence trends are linear and change at a constant rate over the study’s entire period (2). This assumption might not hold when investigating long-term trends or when trends are suspected to be non-linear. Therefore, the sensible approach to analyzing trends in this scenario is by employing a segmented regression analysis wherein more joinpoints are added to the model. This analysis estimates rate changes for different time partitions, known as the segmented annual percentage change (sAPC) (1, 2). For segmented analysis, the percentage of change in incidence rates is assumed constant over specific time intervals defined by joinpoints; yet it might fluctuate over different time partitions. The Joinpoint program users’ guide (3) provides recommendations for the maximum number of joinpoints that could be added to the model based on the number of years covered. The Joinpoint program will select a final model with the optimal number of joinpoints. Yet, the program allows the user to view the results of the other less parsimonious models with a different number of joinpoints.

***Linear regression***

Linear regression models assume a linear relationship between a dependent variable (continuous) and an independent variable (continuous or categorical). Several approaches could be used to fit linear regression models, such as the least-squares and maximum-likelihood estimation techniques (4). Linear regression assumes the dependent variable to have a conditional normal error structure (5). In measuring time trends, the incidence rate is regressed on time, and the slope of this model is transformed through a specific formula $(\left( e^{b}-1 \right)x100)$ to calculate the annual percentage change (APC)(2).

***Generalized linear models (GLM)***

GLM is a class of modeling that generalizes linear regression models allowing for different types of dependent variables like binary, ordinal, nominal, or count data. It also assumes that the errors in the dependent variable have other than a normal distribution, such as binomial or Poisson distribution (5). Parameters in these models are estimated by the maximum likelihood method. GLM doesn’t assume a linear relationship between the dependent and predictor variable. Yet, it allows the transformation of the dependent variable (through the link function), which can linearize the relationship (5).

*Poisson regression*

Poisson regression is a type of GLM used to model count data by assuming that the dependent variable has a Poisson distribution. In this type of modeling, a regression line is fitted to the dependent variable’s natural logarithm (6). In measuring trends, diagnosis year is included in the Poisson regression model as a regressor (independent) variable. The regression coefficient of time represents the incidence rate ratio and could be transformed to present the annual percentage change in incidence rates using the formula $(\left( e^{b}-1 \right)x100)$ (5). A main characteristic of the Poisson distribution is that the mean and variance are assumed equal, a condition known as “equidispersion*”*. When the variance is larger than the mean, “overdispersion” occurs (7). Overdispersion is common and can result in small standard errors and confidence limits, large test statistics, and overestimated significance. Among the most common methods for adjusting overdispersion are the overdispersed Poisson model and the negative binomial model (5).

*Age-period-cohort modeling*

Age-period-cohort modeling is another type of GLM that further investigates incidence trends by describing the simultaneous and independent effect of age (biological processes of aging), birth cohorts (exposures/experiences that vary from one generation to the next), and period (external factors that affect all age groups similarly at a specific calendar time) on cancer incidence (8). Period and cohort effects are usually presented as incidence rate ratios, calculated by comparing the age-specific incidence rate of a given period or cohort group with an arbitrarily chosen referent group. This modeling technique also generates a variety of other parameters providing a comprehensive examination of trends (9).

**References**

1. Kim HJ, Fay MP, Feuer EJ, Midthune DN. Permutation tests for joinpoint regression with applications to cancer rates. Statistics in medicine. 2000;19(3):335-51.

2. Clegg LX, Hankey BF, Tiwari R, Feuer EJ, Edwards BK. Estimating average annual per cent change in trend analysis. Stat Med. 2009;28(29):3670-82.

3. Joinpoint Help Manual 4.8.0.1. *National Cancer Institute, 2020,*  Available from: <https://surveillance.cancer.gov/joinpoint/Joinpoint_Help_4.8.0.1.pdf>.

4. Yan X, Su X. Linear regression analysis: theory and computing: World Scientific; 2009.

5. Coxe S, West SG, Aiken LS. The analysis of count data: a gentle introduction to poisson regression and its alternatives. J Pers Assess. 2009;91(2):121-36.

6. Hayat MJ, Higgins M. Understanding Poisson Regression. Journal of Nursing Education. 2014;53(4):207-15.

7. Berk R, MacDonald JM. Overdispersion and Poisson Regression. Journal of Quantitative Criminology. 2008;24(3):269-84.

8. Rosenberg PS, Anderson WF. Age-period-cohort models in cancer surveillance research: ready for prime time? Cancer Epidemiol Biomarkers Prev. 2011;20(7):1263-8.

9. Murphy CC, Yang YC. Use of age-period-cohort analysis in cancer epidemiology research. Curr Epidemiol Rep. 2018;5(4):418-31.
