# Supplementary material for: Statistical methods for measuring trends in colorectal cancer incidence in registries: A systematic review
Source: Front Oncol. 2022 Nov 30;12:1049486. doi: 10.3389/fonc.2022.1049486 (PMC9748480; doi:10.3389/fonc.2022.1049486)
Supplement: Supplementary file 1 [file DataSheet_1.zip › Table (1).DOCX]

**Supplementary Table 1**

| **Table 1.** Title/Abstract screening inter-reviewer agreement rate calculation (κ statistic) | | | | |
| --- | --- | --- | --- | --- |
|  | | AA* | | |
|  |  | Included | Excluded | Total |
| NA* | Included | 234 (a) | 5 (b) | 239 |
|  | Excluded | 15 (c) | 2652(d) | 2667 |
|  | Total | 249 | 2657 | 2906 |

Alaa

$$p_{0}=\frac{\left( a+d \right)}{a+d+b+c}=\frac{\left( 234+2649 \right)}{2906}=99\%$$

$$p_{e}=\left[ \left( \frac{a+b}{n} \right)* \left( \frac{a+c}{n} \right) \right]+\left[ \left( \frac{c+d}{n} \right)*\left( \frac{b+d}{n} \right) \right]=$$

$$\left[ \left( \frac{239}{2906} \right)* \left( \frac{249}{2906} \right) \right]+\left[ \left( \frac{2667}{2906} \right)*\left( \frac{2657}{2906} \right) \right]=84\%$$

$$\kappa=\frac{\left( p_{0}-p_{e} \right)}{\left( 1-p_{e} \right)}=\frac{\left( 99\%-84\% \right)}{\left( 1-84\% \right)}=94\%$$
