# Supplementary material for: Statistical methods for measuring trends in colorectal cancer incidence in registries: A systematic review
Source: Front Oncol. 2022 Nov 30;12:1049486. doi: 10.3389/fonc.2022.1049486 (PMC9748480; doi:10.3389/fonc.2022.1049486)
Supplement: Supplementary file 1 [file DataSheet_1.zip › Table (2).DOCX]

**Supplementary Tables 2.1, 2.2**

Quality assessment (P.1-6)

|  | | | |
| --- | --- | --- | --- |
| Table 2.1 Quality appraisal checklist | | | |
| Item | **Yes** | **No** | **Unclear** |
| 1. Were the aims/objectives of the study clear? * |  | |  |
| 1. Was the study design appropriate for the stated aim(s)? * |  | |  |
| 1. Was the sample size adequate? § |  | |  |
| 1. Were the study subjects and setting described in detail? § |  | | |
| 1. Were valid methods used for the identification of the condition? § |  | | |
| 1. Were the risk factor and outcome variables measured correctly using instruments/measurements that had been trialed, piloted, or published previously?* |  | | |
| 1. Was there an appropriate statistical analysis? § |  | | |
| 1. Is it clear what was used to determine statistical significance and/or precision estimates? (e.g., p values, CIs)? * |  | | |
| 1. Were the methods (including statistical methods) sufficiently described to enable them to be repeated? * |  | | |
| 1. Were the limitations of the study discussed? * |  | | |

**§** Item from the Joanna Briggs Institute Prevalence Critical Appraisal Tool

*Item from the Appraisal tool for Cross-Sectional Studies

| **Table 2.2** Quality assessment. Studies are sorted in order from the lowest to highest quality | | | | | | | | | | | |
| --- | --- | --- | --- | --- | --- | --- | --- | --- | --- | --- | --- |
| **First author and year** | **Clear aim and objectives** | **Appropriate study design** | **Adequate sample size** | **Description of study subjects and setting** | **Valid methods to identify the condition*** | **Outcome variables were measured correctly using instruments/measurements that had been published previously** | **Appropriate statistical analysis*** | **Determine statistical significance and/or precision estimates** | **Methods sufficiently described enabling reproduction** | **The limitations of the study discussed** | **Total** |
| Al Dahhan 2018 *(19)* | √ | √ | √ | √ | X | √ | X | X | X | X | 5 |
| Klugarova 2019 *(55)* | √ | √ | √ | √ | X | √ | X | X | X | X | 5 |
| Klimczak 2011 *(73)* | √ | √ | √ | √ | X | √ | X | X | X | X | 5 |
| Mosli 2012 *(102)* | √ | √ | √ | √ | X | √ | X | X | X | X | 5 |
| Mosli 2012 *(103)* | √ | √ | √ | √ | X | √ | X | X | X | X | 5 |
| Pescatore 2013 *(115)* | √ | √ | √ | √ | X | √ | X | X | X | X | 5 |
| Palmieri 2013 *(123)* | √ | √ | √ | √ | X | √ | X | X | X | X | 5 |
| Baniasadi 2015 *(26)* | √ | √ | √ | √ | X | √ | X | X | X | X | 5 |
| Zhabagin 2015 *(154)* | √ | √ | √ | √ | X | √ | X | X | X | X | 5 |
| Bailey 2015 *(25)* | √ | √ | √ | √ | X | √ | X | √ | X | X | 6 |
| Crocetti 2010 *(44)* | √ | √ | √ | √ | X | √ | X | √ | X | X | 6 |
| Hasanpour-Heidari 2019 *(52)* | √ | √ | √ | √ | X | √ | X | √ | X | X | 6 |
| Lemmens 2010 *(53)* | √ | √ | √ | √ | X | √ | X | √ | X | X | 6 |
| Nooyi 2011 *(87)* | √ | √ | √ | √ | X | √ | X | √ | X | X | 6 |
| Koblinski 2019 *(93)* | √ | √ | √ | √ | X | √ | X | √ | X | X | 6 |
| Rahman 2015 *(98)* | √ | √ | √ | √ | X | √ | X | √ | X | X | 6 |
| Araghi 2018 *(20)* | √ | √ | √ | √ | X | √ | X | X | X | √ | 6 |
| Bhurgri 2011 *(27)* | √ | √ | √ | √ | √ | √ | X | X | X | X | 6 |
| Winther 2016 *(29)* | √ | √ | √ | √ | √ | √ | X | X | X | X | 6 |
| Chatterjee 2015 *(35)* | √ | √ | √ | √ | X | √ | X | X | X | √ | 6 |
| Alsanea 2015 *(36)* | √ | √ | √ | √ | X | √ | X | X | X | √ | 6 |
| Chen 2012 *(38)* | √ | √ | √ | √ | X | √ | X | X | X | √ | 6 |
| Koblinski 2018 *(56)* | √ | √ | √ | √ | X | √ | X | X | X | √ | 6 |
| Chong 2015 *(42)* | √ | √ | √ | √ | X | √ | X | X | X | √ | 6 |
| Kelly 2012 *(60)* | √ | √ | √ | √ | √ | √ | X | X | X | X | 6 |
| Fowler 2018 *(66)* | √ | √ | √ | √ | X | √ | X | X | X | √ | 6 |
| Merrill 2011 *(72)* | √ | √ | √ | √ | √ | √ | X | X | X | X | 6 |
| Davis 2011 *(91)* | √ | √ | √ | √ | X | √ | X | X | X | √ | 6 |
| Murphy 2017 *(108)* | √ | √ | √ | √ | X | √ | X | X | X | √ | 6 |
| Murphy 2018 *(116)* | √ | √ | √ | √ | X | √ | X | X | X | √ | 6 |
| Zhu 2017 *(122)* | √ | √ | √ | √ | X | √ | X | X | X | √ | 6 |
| Nowicki 2018 *(125)* | √ | √ | √ | √ | √ | √ | X | X | X | X | 6 |
| Oppelt 2019 *(127)* | √ | √ | √ | √ | X | √ | X | X | X | √ | 6 |
| Murphy 2019 *(128)* | √ | √ | √ | √ | X | √ | X | X | X | √ | 6 |
| Keum 2014 *(134)* | √ | √ | √ | √ | X | √ | X | X | X | √ | 6 |
| Ugarte 2012 *(142)* | √ | √ | √ | √ | √ | √ | X | X | X | X | 6 |
| Yee 2010 *(151)* | √ | √ | √ | √ | √ | √ | X | X | X | X | 6 |
| Ohri 2020 *(160)* | √ | √ | √ | √ | X | √ | X | X | X | √ | 6 |
| Garcia 2018 *(82)* | √ | √ | √ | √ | X | √ | X | X | X | √ | 6 |
| Domati 2014 *(92)* | √ | √ | √ | √ | X | √ | X | √ | X | X | 6 |
| Reggiani-Bonetti 2013 *(124)* | √ | √ | √ | √ | X | √ | X | √ | X | X | 6 |
| Abdifard 2013 *(17)* | √ | √ | √ | √ | √ | √ | X | √ | X | X | 7 |
| Chauvenet 2011 *(37)* | √ | √ | √ | √ | √ | √ | X | √ | X | X | 7 |
| Abreu 2010 *(18)* | √ | √ | √ | √ | √ | √ | X | √ | X | X | 7 |
| Ashktorab 2016 *(22)* | √ | √ | √ | √ | X | √ | X | √ | X | √ | 7 |
| Clarke 2014 *(43)* | √ | √ | √ | √ | √ | √ | X | √ | X | X | 7 |
| Ellis 2018 *(48)* | √ | √ | √ | √ | X | √ | X | √ | X | √ | 7 |
| Eser 2018 *(49)* | √ | √ | √ | √ | √ | √ | X | √ | X | X | 7 |
| May 2017 *(54)* | √ | √ | √ | √ | X | √ | X | √ | X | √ | 7 |
| Missaoui 2011 *(59)* | √ | √ | √ | √ | √ | √ | X | √ | X | X | 7 |
| Meester 2019 *(67)* | √ | √ | √ | √ | X | √ | X | √ | X | √ | 7 |
| Li 2017 *(68)* | √ | √ | √ | √ | X | √ | X | √ | X | √ | 7 |
| McDevitt 2017 *(79)* | √ | √ | √ | √ | √ | √ | X | √ | X | X | 7 |
| Khachfe 2019 *(80)* | √ | √ | √ | √ | X | √ | X | √ | X | √ | 7 |
| Brenner 2017 *(83)* | √ | √ | √ | √ | √ | √ | X | √ | X | X | 7 |
| Augustus 2018 *(90)* | √ | √ | √ | √ | X | √ | X | √ | X | √ | 7 |
| Siegel 2017 *(96)* | √ | √ | √ | √ | √ | √ | X | √ | X | X | 7 |
| Savijarvi 2019 *(97)* | √ | √ | √ | √ | X | √ | X | √ | X | √ | 7 |
| Van Beck 2018 *(100)* | √ | √ | √ | √ | X | √ | X | √ | X | √ | 7 |
| Russo 2019 *(104)* | √ | √ | √ | √ | √ | √ | X | √ | X | X | 7 |
| Perdue 2014 *(107)* | √ | √ | √ | √ | X | √ | X | √ | X | √ | 7 |
| Siegel 2020 *(110)* | √ | √ | √ | √ | √ | √ | X | √ | X | X | 7 |
| Shin 2012 *(111)* | √ | √ | √ | √ | √ | √ | X | √ | X | X | 7 |
| Sung 2019 *(119)* | √ | √ | √ | √ | X | √ | X | √ | X | √ | 7 |
| Rafiemanesh 2016 *(120)* | √ | √ | √ | √ | √ | √ | X | √ | X | X | 7 |
| Siegel 2014 *(130)* | √ | √ | √ | √ | √ | √ | X | √ | X | X | 7 |
| Rejali 2018 *(132)* | √ | √ | √ | √ | √ | √ | X | √ | X | X | 7 |
| Sarakarn 2017 *(133)* | √ | √ | √ | √ | √ | √ | X | √ | X | X | 7 |
| Singh 2014 *(135)* | √ | √ | √ | √ | X | √ | X | √ | X | √ | 7 |
| Thuraisingam 2017 *(140)* | √ | √ | √ | √ | X | √ | X | √ | X | √ | 7 |
| Ullah 2018 *(143)* | √ | √ | √ | √ | X | √ | X | √ | X | √ | 7 |
| Wan Ibrahim 2020 *(144)* | √ | √ | √ | √ | X | √ | X | √ | X | √ | 7 |
| Wen 2018 *(148)* | √ | √ | √ | √ | √ | √ | X | √ | X | X | 7 |
| Yeo 2017 *(152)* | √ | √ | √ | √ | X | √ | X | √ | X | √ | 7 |
| Zhang 2018 *(155)* | √ | √ | √ | √ | X | √ | X | √ | X | √ | 7 |
| Oliphant 2011 *(106)* | √ | √ | √ | √ | √ | √ | X | X | X | √ | 7 |
| Abdifard 2016 *(16)* | √ | √ | √ | √ | √ | √ | X | √ | X | X | 7 |
| Brenner 2016 *(30)* | √ | √ | √ | √ | √ | √ | X | X | X | √ | 7 |
| Carroll 2019 *(33)* | √ | √ | √ | √ | √ | √ | X | X | X | √ | 7 |
| Chernyavskiy 2019 *(40)* | √ | √ | √ | √ | X | √ | X | √ | X | √ | 7 |
| Chittleborough 2020 *(41)* | √ | √ | √ | √ | X | √ | X | √ | X | √ | 7 |
| Dehghani 2019 *(46)* | √ | √ | √ | √ | √ | √ | X | √ | X | X | 7 |
| Feletto 2019 *(51)* | √ | √ | √ | √ | X | √ | X | √ | X | √ | 7 |
| Gandhi 2017 *(62)* | √ | √ | √ | √ | X | √ | X | √ | X | √ | 7 |
| McClements 2012 *(64)* | √ | √ | √ | √ | √ | √ | X | X | X | √ | 7 |
| Khiari 2017 *(74)* | √ | √ | √ | √ | √ | √ | X | √ | X | X | 7 |
| Meza 2010 *(77)* | √ | √ | √ | √ | √ | √ | X | X | X | √ | 7 |
| Jafri 2013 *(78)* | √ | √ | √ | √ | √ | √ | X | X | X | √ | 7 |
| Meyer 2010 *(81)* | √ | √ | √ | √ | X | √ | X | √ | X | √ | 7 |
| Fedewa 2019 *(85)* | √ | √ | √ | √ | X | √ | X | √ | X | √ | 7 |
| Nfonsam 2015 *(99)* | √ | √ | √ | √ | √ | √ | X | X | X | √ | 7 |
| Stock 2012 *(136)* | √ | √ | √ | √ | √ | √ | X | X | X | √ | 7 |
| Thirunavukarasu 2010 *(139)* | √ | √ | √ | √ | √ | √ | X | X | X | √ | 7 |
| Wang 2019 *(147)* | √ | √ | √ | √ | √ | √ | X | X | X | √ | 7 |
| Yoon 2015 *(153)* | √ | √ | √ | √ | √ | √ | X | X | X | √ | 7 |
| Zhu 2013 *(157)* | √ | √ | √ | √ | √ | √ | X | X | X | √ | 7 |
| Melnitchouk 2018 *(86)* | √ | √ | √ | √ | X | √ | X | √ | X | √ | 7 |
| Al-Zalabani 2020 *(89)* | √ | √ | √ | √ | X | √ | X | √ | X | √ | 7 |
| Araghi 2019 *(21)* | √ | √ | √ | √ | √ | √ | X | √ | X | √ | 8 |
| Austin 2014 *(23)* | √ | √ | √ | √ | √ | √ | X | √ | X | √ | 8 |
| Aziz 2015 *(24)* | √ | √ | √ | √ | √ | √ | X | √ | X | √ | 8 |
| Boyce 2016 *(28)* | √ | √ | √ | √ | √ | √ | X | √ | X | √ | 8 |
| Brouwer 2018 *(31)* | √ | √ | √ | √ | √ | √ | X | √ | X | √ | 8 |
| Caldarella 2013 *(32)* | √ | √ | √ | √ | √ | √ | X | √ | X | √ | 8 |
| Chambers 2020 *(34)* | √ | √ | √ | √ | √ | √ | X | √ | X | √ | 8 |
| Cheng 2011 *(39)* | √ | √ | √ | √ | √ | √ | X | √ | X | √ | 8 |
| Edwards 2010 *(47)* | √ | √ | √ | √ | √ | √ | X | √ | X | √ | 8 |
| Exarchakou 2019 *(50)* | √ | √ | √ | √ | √ | √ | X | √ | X | √ | 8 |
| Martinsen 2016 *(57)* | √ | √ | √ | √ | √ | √ | X | √ | X | √ | 8 |
| Giddings 2012 *(58)* | √ | √ | √ | √ | √ | √ | X | √ | X | √ | 8 |
| Loomans-Kropp 2019 *(61)* | √ | √ | √ | √ | √ | √ | X | √ | X | √ | 8 |
| Lopez-Abente 2010 *(63)* | √ | √ | √ | √ | √ | √ | X | √ | X | √ | 8 |
| Ladabaum 2014 *(65)* | √ | √ | √ | √ | √ | √ | X | √ | X | √ | 8 |
| Lee 2019 *(71)* | √ | √ | √ | √ | √ | √ | X | √ | X | √ | 8 |
| Jandova 2016 *(75)* | √ | √ | √ | √ | √ | √ | X | √ | X | √ | 8 |
| Li; Lin 2017 *(76)* | √ | √ | √ | √ | √ | √ | X | √ | X | √ | 8 |
| Brenner 2019 *(84)* | √ | √ | √ | √ | √ | √ | X | √ | X | √ | 8 |
| Vuik 2019 *(94)* | √ | √ | √ | √ | √ | √ | X | √ | X | √ | 8 |
| Sheneman 2017 *(105)* | √ | √ | √ | √ | √ | √ | X | √ | X | √ | 8 |
| Vardanjani 2018 *(113)* | √ | √ | √ | √ | √ | √ | X | √ | X | √ | 8 |
| Siegel; Fedewa 2017 *(114)* | √ | √ | √ | √ | √ | √ | X | √ | X | √ | 8 |
| Siegel 2012 *(117)* | √ | √ | √ | √ | √ | √ | X | √ | X | √ | 8 |
| Sierra 2016 *(121)* | √ | √ | √ | √ | √ | √ | X | √ | X | √ | 8 |
| Phipps 2012 *(126)* | √ | √ | √ | √ | √ | √ | X | √ | X | √ | 8 |
| Innos 2018 *(129)* | √ | √ | √ | √ | √ | √ | X | √ | X | √ | 8 |
| Sia 2014 *(131)* | √ | √ | √ | √ | √ | √ | X | √ | X | √ | 8 |
| Sun 2020 *(137)* | √ | √ | √ | √ | √ | √ | X | √ | X | √ | 8 |
| Tawadros 2015 *(138)* | √ | √ | √ | √ | √ | √ | X | √ | X | √ | 8 |
| Troeung 2017 *(141)* | √ | √ | √ | √ | √ | √ | X | √ | X | √ | 8 |
| Wang 2017 *(145)* | √ | √ | √ | √ | √ | √ | X | √ | X | √ | 8 |
| Wang; de Grubb 2017 *(146)* | √ | √ | √ | √ | √ | √ | X | √ | X | √ | 8 |
| Wessler 2010 *(149)* | √ | √ | √ | √ | √ | √ | X | √ | X | √ | 8 |
| Zhou 2015 *(156)* | √ | √ | √ | √ | √ | √ | X | √ | X | √ | 8 |
| Zorzi 2019 *(158)* | √ | √ | √ | √ | √ | √ | X | √ | X | √ | 8 |
| Zorzi 2015 *(159)* | √ | √ | √ | √ | √ | √ | X | √ | X | √ | 8 |
| Crosbie 2018 *(45)* | √ | √ | √ | √ | √ | √ | X | √ | X | √ | 8 |
| Jayarajah 2020 *(69)* | √ | √ | √ | √ | √ | √ | X | √ | X | √ | 8 |
| Katsidzira 2016 *(70)* | √ | √ | √ | √ | √ | √ | X | √ | X | √ | 8 |
| Shafqat 2015 *(95)* | √ | √ | √ | √ | √ | √ | X | √ | X | √ | 8 |
| Shah 2012 *(109)* | √ | √ | √ | √ | √ | √ | X | √ | X | √ | 8 |
| Patel 2016 *(112)* | √ | √ | √ | √ | √ | √ | X | √ | X | √ | 8 |
| Siegel; Medhanie 2019 *(118)* | √ | √ | √ | √ | √ | √ | X | √ | X | √ | 8 |
| Wu 2018 *(150)* | √ | √ | √ | √ | √ | √ | X | √ | X | √ | 8 |
| Siegel 2019 *(88)* | √ | √ | √ | √ | √ | √ | X | √ | X | √ | 8 |
| Wong 2020 *(101)* | √ | √ | √ | √ | √ | √ | X | √ | X | √ | 8 |

***Explanation of indicators:**

- **Valid methods to identify the condition**: This indicator assessed if the study clearly reported the classification system used to assess colorectal cancer and the site codes included in the analysis.
- **Appropriate statistical analysis**: This indicator assessed if the study clearly reported the numerator and denominator data in incidence calculation and described the analytical methods employed in detail.
