# Supplementary material for: Statistical methods for measuring trends in colorectal cancer incidence in registries: A systematic review
Source: Front Oncol. 2022 Nov 30;12:1049486. doi: 10.3389/fonc.2022.1049486 (PMC9748480; doi:10.3389/fonc.2022.1049486)
Supplement: Supplementary file 1 [file DataSheet_1.zip › Table (3).DOCX]

**Supplementary Table 3.** Data extraction sheet (P.1-13)

|  | First author and year | Country | Main outcomes | Observation period | Method(s) for calculating incidence trends | Reporting model fit statistics  (Yes/NR/NA) | Software |
| --- | --- | --- | --- | --- | --- | --- | --- |
|  | Abdifard 2016 *(16)* | Iran | Incidence | 2000-2009 | Visual summary + Poisson regression ^(P2)^ | NR | Stata |
|  | Abdifard 2013 *(17)* | Iran | Incidence | 2000–2005 | Visual summary + Poisson regression ^(P2)^ | NR | Stata |
|  | Abreu 2010 *(18)* | Portugal | Incidence, survival | 1995–2004 | Visual summary + Poisson regression ^(P1)^ | NR | SPSS |
|  | Al Dahhan 2018 *(19)* | Iraq | Incidence | 2002–2011 | Visual summary | NA | Not reported |
|  | Araghi 2018 *(20)* | USA | Incidence, projection | 1973–2014 | Visual summary + Age-period-cohort modelling ^(APC7,10)^ | NR | R  (Nordpred) |
|  | Araghi 2019 *(21)* | Seven high income countries | Incidence | 2008–2012/2009–2013/ 2010–2014 | Visual summary + Join point regression +Age-period-cohort modelling ^(APC7,9,10)^ | Yes (method only)^M1,3^ | APCfit in Stata |
|  | Ashktorab 2016 *(22)* | USA | Incidence | 2000–2012 | Visual summary + Poisson Regression ^(P1)^ | NR | Not reported |
|  | Austin 2014 *(23)* | USA | Incidence | 1998–2009 | visual summary + Linear regression analysis (weighted-least squares method, log-linear model)^L1^ | NR | Stata |
|  | Aziz 2015 *(24)* | USA | Incidence | 1995–2010 | Visual summary + Linear regression analysis ^L1^ | NR | SAS |
|  | Bailey 2015 *(25)* | USA | Incidence | 1975–2010 | Visual summary + Linear regression analysis (weighted-least squares method, log- model) ^L1^ | NR | SEER Stat |
|  | Baniasadi 2015 *(26)* | Iran | Incidence | 2003–2013 | Visual summary + Linear regression analysis ^L3^ | NR | MINITAB |
|  | Bhurgri 2011 *(27)* | Pakistan | Incidence, gender and clinical distribution | 1995–1997/ 1998–2002 | Visual summary | NA | SPSS |
|  | Boyce 2016 *(28)* | Australia | Incidence, clinical and demographic features, survival | 2001-2008 | Visual summary + Poisson regression ^(P1)^ | NR | STATA/SAS |
|  | Winther 2016 *(29)* | Denmark | Incidence, prevalence, survival, mortality | 1980–2012 | Visual summary + Relative change | NA | Not reported |
|  | Brenner 2016 *(30)* | Germany | Incidence, mortality | 2003–2012 | Visual summary + Relative change | NA | Not reported |
|  | Brouwer 2018 *(31)* | Netherlands | Incidence, mortality, treatment, survival | 1989–2014 | Visual summary + Average annual percentage of change analysis (method not specified) | NR | STATA/SAS/SPSS |
|  | Caldarella 2013 *(32)* | Italy | Incidence | 1985–2005 | Visual summary+ Linear regression analysis (weighted-least squares method) ^L1^ | NR | Not reported |
|  | Carroll 2019 *(33)* | USA | Incidence, survival | 1973–2013 | Poisson regression ^(P1)^ | NR | Not reported |
|  | Chambers 2020 *(34)* | United Kingdom | Incidence | 1974–2015 | Visual summary + Join point regression + Age-period-cohort modelling ^(APC2,7)^ | Yes (method and assessment results)^M1,4^ | Joinpoint, Age-Period-Cohort web tool (NCI) |
|  | Chatterjee 2015 *(35)* | USA | Incidence, screening prevalence, CRC risk factors | 2000–2009 | Visual summary + Annual percentage of change analysis (method not specified) | NR | Not reported |
|  | Alsanea 2015 *(36)* | Saudi Arabia | Incidence, survival, demographic features | 1994–2010 | Visual summary | NA | Not reported |
|  | Chauvenet 2011 *(37)* | France | Incidence | 1976–2005 | Visual summary + Poisson regression^(P1)^ + Age-cohort modelling ^(APC1,12)^ | NR | Stata |
|  | Chen 2012 *(38)* | Taiwan | Incidence | 1988–2007 | Visual summary | NA | SPSS |
|  | Cheng 2011 *(39)* | USA | Incidence | 1976–2005 | Visual summary + Relative change + Annual percentage of change analysis (method not specified) | NR | SEER Stat |
|  | Chernyavskiy 2019 *(40)* | USA | Incidence | 2000–2014 | Age-period-cohort modelling ^(APC1,2,7)^ | Yes (method and assessment results)^M6^ | R (Brms) |
|  | Chittleborough 2020 *(41)* | New Zealand, Sweden, and Scotland | Incidence | 1995–2012/1970–2014/ 1990–2014 | Visual summary + Poisson regression ^(P1)^ + Linear regression analysis ^L2^ | NR | R |
|  | Chong 2015 *(42)* | Brunei Darussalam | Incidence | 1991 and 2014 | Visual summary | NA | SPSS |
|  | Clarke 2014 *(43)* | Ireland | Incidence, age and stage distribution, treatment, mortality, survival | 1994-2010 | Visual summary + Join point regression | Yes (method only)^M1^ | Joinpoint |
|  | Crocetti 2010 *(44)* | Italy | Incidence | 1985–2005 | Visual summary +  regression | NR | Joinpoint |
|  | Crosbie 2018 *(45)* | USA | Incidence, demographic and clinical features | 1979–2014 | Visual summary + Join point regression | Yes (method only)^M1^ | Joinpoint |
|  | Dehghani 2019 *(46)* | Iran | Incidence | 2003–2010 | Visual summary + Poisson regression ^(P3)^ | NR | Microsoft Excel |
|  | Edwards 2010 *(47)* | USA | Incidence, mortality, survival, projection | 1975–2006 | Visual summary + Join point regression | Yes (method only)^M1^ | Joinpoint |
|  | Ellis 2018 *(48)* | USA | Incidence | 1990–2014 | Visual summary + Join point regression | Yes (method and assessment results) ^M1^ | Joinpoint |
|  | Eser 2018 *(49)* | Cyprus, Jordan, Israel, and İzmir,Turkey | Incidence | 2005–2010 | Visual summary + Join point regression | NR | Joinpoint |
|  | Exarchakou 2019 *(50)* | England | Incidence | 1971–2014 | Visual summary + Join point regression | Yes (method only)^M1^ | Joinpoint |
|  | Feletto 2019 *(51)* | Australia | Incidence | 1982–2014 | Visual summary + Join point regression + Age-period-cohort modelling ^(APC2,7)^ | Yes (method only)^M1^ | Joinpoint, Age-Period-Cohort web tool (NCI) |
|  | Hasanpour-Heidari 2019 *(52)* | Iran | Incidence | 2004–2013 | Visual summary + Join point regression | Yes (method only)^M1^ | Joinpoint |
|  | Lemmens 2010 *(53)* | Netherlands | Incidence, stage distribution, treatment, mortality, survival | 1975–2007 | Visual summary | NA | Not reported |
|  | May 2017 *(54)* | USA | Incidence, stage distribution | 1975–2012 | Visual summary + Join point regression | Yes (method only)^M1^ | Joinpoint |
|  | Klugarova 2019 *(55)* | Czech Republic | Incidence, prevalence, mortality, treatment, survival | 1982–2016 | Visual summary | NA | Not reported |
|  | Koblinski 2018 *(56)* | USA | Incidence | 2000–2010 | Visual summary + Linear regression analysis ^L1^ | NR | SPSS |
|  | Martinsen 2016 *(57)* | USA | Incidence, mortality, survival | 1990–2012 | Visual summary + Join point regression | NR | Joinpoint |
|  | Giddings 2012 *(58)* | USA | Incidence | 1988–2007 | Visual summary + Join point regression | NR | Joinpoint |
|  | Missaoui 2011 *(59)* | Tunisia | Incidence | 1993–2007 | Visual summary + Linear regression analysis (log-linear model) ^L1^ | NR | Not reported |
|  | Kelly 2012 *(60)* | USA | Incidence | 2005–2009 | Visual summary | NA | Not reported |
|  | Loomans-Kropp 2019 *(61)* | USA | Incidence, mortality | 1980–2016 | Visual summary + Join point regression | Yes (method only)^M1^ | Joinpoint |
|  | Gandhi 2017 *(62)* | New Zealand | Incidence | 1995–2012 | Visual summary + Poisson regression ^(P1)^ | Yes (method and assessment results)^M2^ | Not reported |
|  | Lopez-Abente 2010 *(63)* | Spain | Incidence, mortality | 1975–1993/2000–2004 | Visual summary + Poisson regression^(P1)^ (Change-point model/ Age-period-cohort modelling^(APC1,9)^) | NR | R |
|  | McClements 2012 *(64)* | United Kingdom | Incidence, stage distribution, mortality | 1982–2006 | Visual summary | NA | SPSS + STATA |
|  | Ladabaum 2014 *(65)* | USA | Incidence | 1990–2004 | Visual summary + Join point regression | Yes (method only)^M1^ | Not reported |
|  | Fowler 2018 *(66)* | USA | Incidence, mortality | 1991–2010 | Visual summary + LOESS method to generate nonparametric local regression smoothing | NR | SAS |
|  | Meester 2019 *(67)* | USA | Incidence, stage distribution | 1975–2015 | Visual summary + Join point regression | NR | Joinpoint |
|  | Li 2017 *(68)* | China | Incidence | 1998–2012 | Visual summary + Join point regression | NR | Joinpoint |
|  | Jayarajah 2020 *(69)* | Sri Lanka | Incidence, clinical features | 2001–2010 | Visual summary + Join point regression | Yes (assessment results only) | Joinpoint |
|  | Katsidzira 2016 *(70)* | Zimbabwe | Incidence, demographic and clinical features | 2003–2012 | Visual summary + Join point regression | NR | Joinpoint |
|  | Lee 2019 *(71)* | Taiwan | Incidence, survival, mortality | 1984–2013 | Visual summary + Poisson regression ^(P1)^ + Age-period-cohort modelling^(APC2,11)^ | NR | SAS, WinBUGS |
|  | Merrill 2011 *(72)* | USA | Incidence | 2005–2007 | Relative change | NA | Not reported |
|  | Klimczak 2011 *(73)* | Poland | Incidence, prevalence | 1999–2008 | Visual summary | NA | Not reported |
|  | Khiari 2017 *(74)* | Tunisia | Incidence, projection | 1994–2009 | Visual summary + Join point regression | NR | Joinpoint |
|  | Jandova 2016 *(75)* | USA | Incidence, mortality, demographic and clinical features | 1995–2011 | Visual summary + Relative change | NA | SPSS |
|  | Li; Lin 2017 *(76)* | China | Incidence | 2010–2014 | Visual summary + Join point regression | NR | Joinpoint |
|  | Meza 2010 *(77)* | United Kingdom and USA | Incidence | 1973–2006 | Visual summary + Poisson regression^(P1)^ (Age-period-cohort modelling^(APC7)^ | NR | Not reported |
|  | Jafri 2013 *(78)* | USA | Incidence, survival | 1993–2007 | Visual summary + Poisson regression ^(P1)^ + Linear regression analysis (weighted-least squares method) ^L1^ | NR | SAS |
|  | McDevitt 2017 *(79)* | Ireland | Incidence, mortality, survival, anatomical site and stage distribution | 1994–2012 | Join point regression | Yes (method only)^M1^ | Not reported |
|  | Khachfe 2019 *(80)* | Lebanon | Incidence | 2005–2015 | Visual summary + Join point regression | Yes (assessment results only) | Joinpoint |
|  | Meyer 2010 *(81)* | USA | Incidence | 1973–2005 | Visual summary + Join point regression + Linear regression analysis (weighted-least squares method) ^L1^ | NR | SEER Stat + Joinpoint |
|  | Garcia 2018 *(82)* | USA | Incidence | 2001–2014 | Visual summary + Absolute and relative change | NA | Not reported |
|  | Brenner 2017 *(83)* | Canada | Incidence | 1971–2012 | Visual summary + Join point regression + Age-period-cohort modelling^(APC7)^ + Interrupted time-series regression analysis | Yes (method only)^M1^ | Joinpoint, Age-Period-Cohort web tool (NCI) |
|  | Brenner 2019 *(84)* | Canada | Incidence | 1971–2015 | Visual summary + Join point regression+ Age-period-cohort modelling^(APC7)^ | Yes (method only)^M1^ | Joinpoint, Age-Period-Cohort web tool (NCI) |
|  | Fedewa 2019 *(85)* | USA | Incidence/ colonoscopy rate | 2000–2015 | Visual summary + Join point regression +Incidence rate ratios | NR | Joinpoint |
|  | Melnitchouk 2018 *(86)* | Ukraine | Incidence, mortality, treatment, stage distribution | 2000 –2014 | Visual summary + Join point regression | Yes (method only)^M1^ | Joinpoint |
|  | Nooyi 2011 *(87)* | India | Incidence | 1968–2002 | Visual summary + Mean annual percentage change (MAPC) + Poisson regression ^(P1)^ | NR | SAS |
|  | Siegel 2019 *(88)* | Global | Incidence | 2008–2012 | Visual summary + Join point regression | Yes (method only)^M1^ | Not reported |
|  | Al-Zalabani 2020 *(89)* | Saudi Arabia | Population attributable fraction (PAF), Incidence, projection | 1994–2015 | Visual summary + Join point regression | Yes (method and assessment results)^M1^ | Joinpoint |
|  | Augustus 2018 *(90)* | USA | Incidence | 2000–2014 | Visual summary + Join point regression | NR | Joinpoint, R |
|  | Davis 2011 *(91)* | USA | Incidence, age and anatomical site distribution | 1987–2006 | Visual summary + Relative change | NA | Microsoft Excel |
|  | Domati 2014 *(92)* | Italy | Incidence, survival, clinical features | 1986–2008 | Visual summary + Join point regression | Yes (method only)^M1^ | joinpoint |
|  | Koblinski 2019 *(93)* | USA | Incidence, demographic and clinical features | 2000–2010 | Visual summary + linear regression analysis ^L1^ | NR | SPSS |
|  | Vuik 2019 *(94)* | Europe | Incidence, mortality | 1990–2016 | Visual summary + Join point regression | NR | Joinpoint |
|  | Shafqat 2015 *(95)* | USA | Incidence, survival, management | 2000–2011 | Visual summary + Join point regression | NR | Joinpoint |
|  | Siegel 2017 *(96)* | USA | Incidence, mortality, survival, stage distribution, screening prevalence | 2009–2013 | Visual summary + Join point regression | NR | Joinpoint |
|  | Savijarvi 2019 *(97)* | Finland | Incidence | 1976–2014 | Visual summary + Poisson regression ^(P1)^ | NR | Not reported |
|  | Rahman 2015 *(98)* | USA | Incidence, survival | 1992–2009 | Visual summary + Join point regression | NR | Not reported |
|  | Nfonsam 2015 *(99)* | USA | Incidence, mortality, stage distribution | 1995–2010 | Visual summary | NA | SAS |
|  | Van Beck 2018 *(100)* | USA | Incidence, mortality | 1976–2015 | Visual summary+ join point regression | NR | Joinpoint |
|  | Wong 2020 *(101)* | 39 countries | Incidence, mortality | 1980–2016 | Visual summary + Join point regression | NR | Not reported |
|  | Mosli 2012 *(102)* | Saudi Arabia | Incidence, clinical features | 2001–2006 | Visual summary | NA | Microsoft Excel |
|  | Mosli 2012 *(103)* | Saudi Arabia | Incidence, clinical features | 2000–2006 | Visual summary | NA | Microsoft Excel |
|  | Russo 2019 *(104)* | Italy | Incidence | 1999–2015 | Visual summary + Join point regression + Age-period-cohort modelling^(APC7,8,9)^ | NR | Joinpoint, R(macro) |
|  | Sheneman 2017*(105)* | USA | Incidence, survival | 1992–2013 | Visual summary + linear regression analysis (log-linear model) ^L1^ | NR | Microsoft Excel |
|  | Oliphant 2011 *(106)* | United Kingdom | Incidence | 1999 –2007 | Visual summary | NA | STATA |
|  | Perdue 2014 *(107)* | USA | Incidence, mortality | 2005–2009 | Join point regression | Yes (method only)^M1^ | joinpoint |
|  | Murphy 2017 *(108)* | USA | Incidence | 1975–2013 | Visual summary + Relative change | NA | Not reported |
|  | Shah 2012 *(109)* | New Zealand | Incidence | 1981–2004 | Visual summary + Linear regression analysis (weighted-least squares method) ^L1^ + Relative change | NR | SAS |
|  | Siegel 2020 *(110)* | USA | Incidence, mortality, screening prevalence, survival, stage distribution | 2012–2016/1995–2016 | Visual summary + Join point regression | NR | joinpoint |
|  | Shin 2012 *(111)* | Korea | Incidence | 1999–2009 | Visual summary + Linear regression analysis (log-linear model) ^L1^ | NR | R |
|  | Patel 2016 *(112)* | Canada | Incidence, CRC risk factors | 1969–2010 | Visual summary + Join point regression | Yes (method only)^M1^ | Joinpoint |
|  | Vardanjani 2018 *(113)* | Iran | Incidence, prevalence, projection | 2003–2012 | Join point regression | NR | Joinpoint |
|  | Siegel; Fedewa 2017 *(114)* | USA | Incidence | 1974–2013 | Visual summary + Join point regression + Age-period-cohort modelling^(APC1-7,9)^ | Yes (method and assessment results)^M1,5^ | Joinpoint, Age-Period-Cohort web tool (NCI) |
|  | Pescatore 2013 *(115)* | Luxembourg | Incidence, survival, stage distribution | 1990–2009 | Visual summary | NA | Not reported |
|  | Murphy 2018 *(116)* | USA | Incidence | 1975–2014 | Visual summary + Age-period-cohort modelling^(APC7,10)^ | NR | Age-Period-Cohort web tool (NCI) |
|  | Siegel 2012 *(117)* | USA | Incidence | 1992–2008 | Visual summary + Join point regression | Yes (method only)^M1^ | joinpoint |
|  | Siegel; Medhanie 2019 *(118)* | USA | Incidence, CRC risk factors | 1995–2015 | Join point regression | Yes (method only)^M1^ | joinpoint |
|  | Sung 2019 *(119)* | Hong Kong, Korea, Japan, and Taiwan | Incidence | 1995–2014 | Visual summary + Join point regression | Yes (method only)^M1^ | joinpoint |
|  | Rafiemanesh 2016 *(120)* | Iran | Incidence, clinical features | 2003–2008 | Join point regression | NR | joinpoint |
|  | Sierra 2016 *(121)* | Central and South America | Incidence, mortality | 2003–2007 | Visual summary +Annual percentage of change analysis (method not specified) | NR | STATA |
|  | Zhu 2017 *(122)* | China | Incidence, mortality, projection | 2003–2011 | Visual summary | NA | SPSS |
|  | Palmieri 2013 *(123)* | Italy | Incidence, mortality, survival, demographic and clinical features | 1992–2010 | Visual summary | NA | Not reported |
|  | Reggiani-Bonetti 2013 *(124)* | Italy | Incidence, clinical features | 1986–2008 | Visual summary + Join point regression | Yes (method only)^M1^ | Joinpoint |
|  | Nowicki 2018 *(125)* | Poland | Incidence, morbidity, survival | 2006–2011 | Visual summary | NA | Statistica, Microsoft Excel |
|  | Phipps 2012 *(126)* | USA | Incidence, mortality | 1975–2007 | Visual summary + Join point regression | Yes (method only)^M1^ | Joinpoint |
|  | Oppelt 2019 *(127)* | Germany | Incidence | 2008–2014 | Visual summary | NA | SAS |
|  | Murphy 2019 *(128)* | USA | Incidence, survival | 1992–2014 | Relative and absolute change | NA | Not reported |
|  | Innos 2018 *(129)* | Estonia | Incidence, survival | 1995–2014 | Visual summary + Join point regression | NR | Joinpoint |
|  | Siegel 2014 *(130)* | USA | Incidence, survival, mortality, anatomical site and stage distribution | 1975–2010 | Visual summary + Join point regression | NR | Joinpoint |
|  | Sia 2014 *(131)* | Australia | Incidence, anatomical site and histopathology distribution | 2000–2010 | Visual summary + Join point regression + Poisson regression ^(P1)^ | NR | Joinpoint, Stata |
|  | Rejali 2018 *(132)* | Iran | Incidence | 2000–2011 | Visual summary + Join point regression | Yes (method only)^M1^ | Joinpoint |
|  | Sarakarn 2017 *(133)* | Thailand | Incidence | 1989–2012 | Visual summary + Join point regression | Yes (method only)^M1^ | Joinpoint |
|  | Keum 2014 *(134)* | USA | Incidence, mortality | 1975–2009 | Visual summary + Join point regression | NR | Joinpoint |
|  | Singh 2014 *(135)* | USA | Incidence | 1988–2009 | Join point regression | NR | Not reported |
|  | Stock 2012 *(136)* | USA | Cumulative risk | 1978–2007 | Visual summary+ Relative change | NA | Not reported |
|  | Sun 2020 *(137)* | Sweden | Incidence, survival | 1960–2014 | Visual summary + Join point regression | Yes (method only)^M1^ | Joinpoint |
|  | Tawadros 2015 *(138)* | USA | Incidence, clinical features | 1980–2010 | Visual summary + Linear regression analysis (weighted-least squares method) ^L1^ | NR | Not reported |
|  | Thirunavukarasu 2010 *(139)* | USA | Incidence, survival, clinical and demographic features | 1973–2006 | Visual summary | NA | SPSS |
|  | Thuraisingam 2017 *(140)* | USA | Incidence | 2000–2012 | Visual summary+ Relative change | NA | SPSS |
|  | Troeung 2017 *(141)* | Australia | Incidence, mortality, colonoscopy history | 1982–2007 | Visual summary + Join point regression | Yes (method and assessment results)^M1^ | Joinpoint |
|  | Ugarte 2012 *(142)* | Spain | Incidence | 1990–2005 | Visual summary + Bayesian analysis of spatio-temporal conditional autoregressive models | NR | R |
|  | Ullah 2018 *(143)* | Ireland | Incidence, stage distribution | 1994–2012 | Visual summary + Linear regression analysis ^L1^ | NR | SPSS |
|  | Wan Ibrahim 2020 *(144)* | Malaysia | Incidence, mortality, survival, clinical and demographic features | 2007–2017 | Visual summary + Time-series regression analysis | NR | R |
|  | Wang 2017 *(145)* | USA | Incidence, survival, stage distribution | 1995–2010 | Visual summary + Annual percentage of change analysis (method not specified) | NR | SEER Stat |
|  | Wang; de Grubb 2017 *(146)* | USA | Incidence | 1994–2013 | Visual summary + Linear regression analysis (weighted-least squares method) ^L1^ | NR | SEER Stat + SPSS |
|  | Wang 2019 *(147)* | USA | Incidence, factors associated with cancer-specific death | 1988–2013 | Visual summary | NA | SAS |
|  | Wen 2018 *(148)* | China | Incidence | 2012/2000–2015 | Visual summary + Join point regression | NR | Joinpoint |
|  | Wessler 2010 *(149)* | Norfolk, Suffolk, Cambridgeshire (NSC)  (East of England) | Incidence | 1971–2005 | Visual summary + Poisson regression ^(P1)^ (Age-period-cohort modelling) ^(APC1,7)^ | Yes (method and assessment results)^M2,3^ | STATA |
|  | Wu 2018 *(150)* | Shanghai | Incidence, mortality | 1975–2013 | Visual summary + Join point regression + Age-period-cohort modelling^(APC2,3,7)^ | Yes (method only)^M1^ | Joinpoint, Age-Period-Cohort web tool (NCI) |
|  | Yee 2010 *(151)* | Hong Kong | Incidence | 1983–2006 | Visual summary + Relative change | NA | Not reported |
|  | Yeo 2017 *(152)* | USA | Incidence, clinical and demographic features | 2000–2011 | Visual summary + Linear regression analysis ( least squares method) ^L1^ | NR | STATA |
|  | Yoon 2015 *(153)* | Korea | Incidence, mortality, fatality, screening rate | 1999–2012 | Visual summary | NA | Not reported |
|  | Zhabagin 2015 *(154)* | Kazakhstan | Incidence, mortality | 2004–2013 | Visual summary | NA | Not reported |
|  | Zhang 2018 *(155)* | Hong Kong | Incidence | 1983–2012 | Visual summary + Join point regression + Age-period-cohort modelling^(APC1-3,7)^ | Yes (method only)^M1^ | Joinpoint, Age-Period-Cohort web tool (NCI) |
|  | Zhou 2015 *(156)* | China/Guangzhou | Incidence, age and anatomical site distribution | 2000–2011 | Visual summary + Join point regression | NR | Joinpoint |
|  | Zhu 2013 *(157)* | USA | Incidence | 1973–2008 | Visual summary+ Poisson regression^(P1)^ (Age-period-cohort modelling ^(APC9)^) | NR | SAS |
|  | Zorzi 2019 *(158)* | Italy | Incidence, mortality | 2003–2014 | Visual summary + Join point regression | Yes (method only)^M1^ | Joinpoint |
|  | Zorzi 2015 *(159)* | Italy | Incidence | 2000–2008 | Visual summary + Annual percentage of change analysis (method not specified) | NR | Not reported |
|  | Ohri 2020 *(160)* | USA | Incidence | 2000–2014 | Visual summary + Relative change | NA | SEER Stat |

**Abbreviations:** NR: Not reported, NA: Not applicable, CRC: Colorectal cancer, USA: United States of America.

**Presentation of incidence trends:**

1-*Linear regression:* L1: percentage of change; L2: difference per decade; L3: Reporting only model formulae.

2-*Poisson regression:* P1: Study reported trends as a percentage of change or as incidence rate ratio (IRR); P2: Study reported trends as merely the slope of the regression line; P3: Study reported trends by only stating the significance of incidence rate trends.

*3-APC modeling:* APC1: Net drift (age-adjusted annual percentage change); APC2: Local drift (age-specific net annual percentage change); APC3: Longitudinal age curve (Fitted longitudinal age-specific rates in reference cohort adjusted for period deviations); APC4: Cross-sectional age curve (Fitted cross-sectional age-specific rates in reference period p0 adjusted for cohort deviations); APC5.Age, period, and cohort deviations (measure curvature, which describes local changes in trends, independently of the magnitude or direction of the overall trend); APC6.Fitted temporal trends (Fitted rates in reference age group adjusted for cohort deviations); APC7.Period/Cohort rate ration (Ratio of rates in a certain period/cohort relative to reference period/cohort); APC8.Graphical presentation of rates according to age group; APC9.Graphical presentation of trends in age-specific rates by year of birth; APC10.Graphical presentation of trends in age-specific rates by calendar period; APC11.Annual Absolute risk difference in CRC by cohort and age; APC12.Cumulative risk-over the age range (0-74)- of developing CRC according to birth cohorts.

**Model validity assessment:** M1:Permutation test; M2:likelihood ratio tests; M3:Deviance statistics; M4:The squared correlation coefficient (R^2^); M5:Residual analysis; M6:Standard posterior distribution predictive checks (for age-period-cohort modeling).
