# Supplementary material for: Statistical methods for measuring trends in colorectal cancer incidence in registries: A systematic review
Source: Front Oncol. 2022 Nov 30;12:1049486. doi: 10.3389/fonc.2022.1049486 (PMC9748480; doi:10.3389/fonc.2022.1049486)
Supplement: Supplementary file 1 [file DataSheet_1.zip › Table (4).DOCX]

**Supplementary Table 4**

Results Table (P.1-6)

| Table 4. Description of characteristics of included studies | |
| --- | --- |
| Study characteristics | |
| *Country* | N (% out of 145) |
| The United States of America | 58 (40.0) |
| Canada | 3(2.0) |
| Europe | 34 (23.4) |
| Asia | 33 (22.8) |
| Oceania | 6 (4.1) |
| Africa | 3 (2.0) |
| Multiple countries | 8 (5.5) |
| *Main outcomes* (presented here are the three most common outcomes reported in the included studies) | N (% out of 145) |
| Incidence | 144 (99.3) |
| Mortality | 35 (24.1) |
| Survival | 30 (20.7) |
| *Observation period* | N (% out of 145) |
| Less than 10 years | 21 (14.5) |
| 10-19 years | 57 (39.3) |
| 20 years or more | 69 (47.6) |
| Methods used to measure incidence trends | |
| 1. Explanatory methods | ***N (% out of 145)*** |
| Visual summaries. | 135 (93.1) |
| Study reported trends using only visual summaries. | 23 (15.9) |
| Study reported trends as a percentage of change (relative change)   - Trend presented as relative and absolute change - Study reported the formulae to calculate the relative change - Study reported confidence interval estimates - Study reported the significance of trends | 14 (9.7)  2 (14.3% out of 14)  2 (14.3% out of 14)  0  2 (14.3% out of 14) |
| Study reported the incidence trend as the mean annual percentage change calculated via a mathematical equation. | 1 (0.7) |
| Study calculated the incidence trend using the incidence rate ratio. | 1 (0.7) |
| 2. Statistical modeling methods | |
| 1. *Joinpoint regression* | ***N (% out of 145)*** |
| Study analyzed trends using joinpoint regression. | 65 (44.8) |
| *Presentation of trends:* | |
| - Study did not report the percentage of change in trends | 1 (1.5% out of 65) |
| - Study reported only annual percentage of change (APC)   - Study presented one APC over the whole observation period   - Study presented several APCs over different time segments | 37(56.9% out of 65)  15 (40.5% out of 37)  22 (59.4% out of 37) |
| - Study reported only average annual percentage change (AAPC)   - Study stated a clear explanation of AAPC calculation | 11 (16.9% out of 65)  1 (9.1% out of 11) |
| - Study reported APC and AAPC   - Study explicitly stated the difference in calculation between APC and AAPC   - Study stated the difference between APC and AAPC by reporting the number of years covered for each measure   - Study did not state the difference between APC and AAPC | 16 (24.6% out of 65)  4 (25.0% out of 16)  6 (37.5% out of 16)  6 (37.5% out of 16) |
| Software employed for conducting joinpoint regression:   - Joinpoint trend analysis software, National Cancer Institute. | 58 (89.2% out of 65) |
| Study reported information on parameter setting in the joinpoint program:   - Study specified the used modeling method (Grid search or Hudson’s). - Study reported the chosen minimum APC difference worth detecting. - Study reported the minimum number of joinpoints selected. - Study reported the maximum number of joinpoints selected. - Study reported the AAPC segment ranges that were selected. - Study reported the chosen model selection method. - Study reported the method used for estimating confidence intervals. - Study reported the chosen autocorrelated errors option. - Study reported selecting a linear or log-linear model. | ***N (% out of 58)***  0  0  8 (13.8% out of 58)  17(29.3% out of 58)  1(1.7% out of 58)  30(51.7% out of 58)  1(1.7% out of 58)  0  17(29.3% out of 58) |
| 1. *Linear regression models* | ***N (% out of 145)*** |
| Study analyzed trends using linear regression models. | 18 (12.4) |
| Study reported the use of the least-squares method to fit the model. | 1 (5.6% out of 18) |
| Study reported the use of the weighted least-squares method to fit the model. | 8 (44.4 out of 18) |
| Study reported log transformation of the model. | 5 (27.8% out of 18) |
| *Presentation of trends:*   - Percentage of change - Difference per unit of time - Reporting only model formulae with no estimates for trends | 16 (88.9% out of 18)  1 (5.6% out of 18)  1 (5.6% out of 18) |
| Software employed for conducting linear regression:   - SPSS - SAS - SEER Stat - Stata - R - Microsoft Excel - MINITAB | 4 (22.2% out of 18)  3 (16.7% out of 18)  3 (16.7% out of 18)  2 (11.1% out of 18)  2 (11.1% out of 18)  1 (5.6% out of 18)  1 (5.6% out of 18) |
| *Generalized linear models* | |
| C1. Poisson regression | ***N (% out of 145)*** |
| Study analyzed trends using Poisson regression. | 19 (13.1) |
| Study reported the use of Poisson regression to conduct age-period-cohort analysis. | 5 (26.3% out of 19) |
| *Presentation of trends:*   - Study reported trends as incidence rate ratio (IRR) or percentage of change - Study reported trends as merely the slope of the regression line. - Study reported trends by only stating the significance of incidence rate trends. | 16 (84.2% out of 19)  2 (10.5% out of 19)  1 (5.2% out of 19) |
| Consideration for dispersion was reported:   - Overdispersion corrected using negative binomial distribution - No indication of the method used to correct overdispersion | 2 (10.5% out of 19)  1 (5.2% out of 19)  1 (5.2% out of 19) |
| Software employed for conducting Poisson regression:   - Stata - SAS - R - Winbugs - SPSS - Microsoft Excel | 6 (31.5% out of 19)  5 (26.3% out of 19)  2 (10.5% out of 19)  1 (5.2% out of 19)  1 (5.2% out of 19)  1 (5.2% out of 19) |
| C2. Age-Period-Cohort modeling (APCM) | ***N (% out of 145)*** |
| Study analyzed trends using APCM. | 18 (12.4) |
| Study performed only APCM to assess trends. | 1 (0.7) |
| Study performed APCM and joinpoint regression analysis. | 9 (6.2) |
| *Presentation of trends:* | |
| - Period/Cohort rate ratio (Ratio of rates in a certain period/cohort relative to reference period/cohort).* - Reference category for Period/Cohort rate ratio estimation:   - Middle calendar period and birth cohort groups   - Earliest period and cohort groups   - The cohort with the lowest incidence rates | 14 (77.8% out of 18)  9 (64.3% out of 14)  2 (14.3% out of 14)  1 (7.1% out of 14) |
| - Local drift (age-specific net annual percentage change).* | 7 (38.9% out of 18) |
| - Net drift (age-adjusted annual percentage change).* | 6 (33.3% out of 18) |
| - Longitudinal age curve (Fitted longitudinal age-specific rates in reference cohort adjusted for period deviations).* | 3 (16.7% out of 18) |
| - Graphical presentation of trends in age-specific rates by year of birth. | 5 (27.8% out of 18) |
| - Graphical presentation of trends in age-specific rates by calendar period. | 3 (16.7% out of 18) |
| - Cross-sectional age curve (Fitted cross-sectional age-specific rates in reference period adjusted for cohort deviations).* | 1 (5.5% out of 18) |
| - Age, period, and cohort deviations (measure curvature, which describes local changes in trends, independently of the magnitude or direction of the overall trend).* | 1 (5.5% out of 18) |
| - Fitted temporal trends (Fitted rates in reference age group adjusted for cohort deviations).* | 1 (5.5% out of 18) |
| - Graphical presentation of rates according to age group. | 1 (5.5% out of 18) |
| - Annual Absolute risk difference in CRC by cohort and age. | 1(5.5% out of 18) |
| - Cumulative risk-over the age range (0-74)- of developing CRC according to birth cohorts. | 1(5.5% out of 18) |
| Software used to apply APCM:   - Age-Period-Cohort web tool, National cancer institute - R (nordpred, brms, macro) - STATA - SAS - Winbugs | 8 (44.4% out of 18)  4 (22.2% out of 18)  3 (16.7% out of 18)  2 (11.1% out of 18)  1(5.5% out of 18) |
| 1. *Other methods* | ***N (% out of 145)*** |
| - Study reported Time series analysis. | 1 (0.7) |
| - Study reported Interrupted time series analysis. | 1 (0.7) |
| - Study reported Bayesian analysis of spatio-temporal conditional autoregressive models. | 1 (0.7) |
| - Study reported the LOESS method to generate nonparametric local regression smoothing. | 1 (0.7) |
| - Study reported the calculation of APC with no further explanation of the used statistical method. | 6 (4.1) |
| Model validity measures | ***N (% out of 104 studies that used modeling)*** |
| Study reported an assessment of model fitness.  Study reported the results of the model fit assessment. | 39 (37.5)  10 (9.6) |
| *Methods to evaluate the model fit (as reported in studies):*   - Permutation test (for Joinpoint modeling) - likelihood ratio tests (for Poisson modeling) - Deviance statistics (for age-period-cohort modeling) - The squared correlation coefficient (R2) (for Joinpoint modeling) - Residual analysis (for age-period-cohort modeling) - Standard posterior distribution predictive checks (for age-period-cohort modeling) | 34 (32.7)  2 (1.9)  2 (1.9)  1 (0.9)  1 (0.9)  1 (0.9) |
| Software reported for estimating incidence trends | ***N (% out of 145)*** |
| Study reported software information for incidence trend analysis | 111 (76.6) |
| Software reported for estimating incidence trends: | ***N (% out of 111)*** |
| Joinpoint | 58 (52.3) |
| SPSS | 14 (12.6) |
| STATA | 13 (11.7) |
| SAS | 12 (10.8) |
| R | 9 (8.1) |
| Age Period Cohort web tool, National cancer institute (NCI) | 8 (7.2) |
| SEER Stat | 6 (5.4) |
| Microsoft Excel | 6 (5.4) |
| MINITAB | 1 (0.9) |
| WinBUGS | 1 (0.9) |
| Statistica | 1 (0.9) |
