# Supplementary material for: Statistical methods for measuring trends in colorectal cancer incidence in registries: A systematic review
Source: Front Oncol. 2022 Nov 30;12:1049486. doi: 10.3389/fonc.2022.1049486 (PMC9748480; doi:10.3389/fonc.2022.1049486)
Supplement: Supplementary file 1 [file DataSheet_1.zip › Table (5).DOCX]

**Supplementary Table 5**

Joinpoint Regression Analysis (P.1-3)

**Table 5.** Results of joinpoint regression analysis

| First author and year | Study period (Number of years) | Estimated measure* | Joinpoint analysis  (Number of time segments/number of joinpoints) | Providing information on setting parameters in the joinpoint program* |
| --- | --- | --- | --- | --- |
| Araghi 2019 *(20)* | 10 | AAPC | One time segment | Not applicable |
| Chambers 2020 *(34)* | 41 | APC (for various periods) | (2-3 / 1-2) | Yes ^(3,4,6,9)^ |
| Clarke 2014 *(43)* | 17 | APC (for the whole period) | (1/0) | Yes ^(6)^ |
| Crocetti 2010 *(44)* | 21 | APC (for various periods) | (2/1) | No |
| Crosbie 2018 *(45)* | 36 | APC (for various periods) | (1-3/ 0-2) | Yes ^(6)^ |
| Edwards 2010 *(47)* | 32 | APC (for various periods) and AAPC^2^ | (5/ 4) | Yes ^(6)^ |
| Ellis 2018 *(48)* | 25 | Triannual percentage change (for the whole period) | (1/0) | Yes ^(3,4,6)^ |
| Eser 2018 *(49)* | 6 | APC (for the whole period) | (1/0) | No |
| Exarchakou 2019 *(50)* | 44 | APC (for various periods) | (2-3/ 1-2) | Yes ^(4,6,9)^ |
| Feletto 2019 *(51)* | 33 | APC (for various periods) and AAPC^3^ | (1-4/ 0-3) | Yes ^(6)^ |
| Hasanpour-Heidari 2019 *(52)* | 10 | AAPC | One time segment | Yes ^(6)^ |
| May 2017 *(54)* | 38 | APC (for various periods) and AAPC^2^ | (3-5/2-4) | Yes ^(6)^ |
| Martinsen 2016 *(57)* | 23 | APC (for various periods) | (1-2/0-1) | No |
| Giddings 2012 *(58)* | 20 | APC (for the whole period) | (1/0) | Yes ^(3,4,9)^ |
| Loomans-Kropp 2019 *(61)* | 37 | APC (for various periods) | (2-5/1-4) | Yes ^(4,6)^ |
| Ladabaum 2014 *(65)* | 15 | APC (for various periods) | (1-3/0-2) | Not applicable |
| Meester 2019 *(67)* | 49 | AAPC | One time segment | Yes ^(9)^ |
| Li 2017 *(68)* | 15 | APC (for various periods) | (1-2/0-1) | No |
| Jayarajah 2020 *(69)* | 10 | APC (for the whole period) | (1/0) | Yes ^(9)^ |
| Katsidzira 2016 *(70)* | 10 | AAPC | One time segment | No |
| Khiari 2017 *(74)* | 16 | APC (for the whole period) | (1/0) | No |
| Li; Lin 2017 *(76)* | 5 | AAPC | One time segment | No |
| McDevitt 2017 *(79)* | 18 | APC (for the whole period) | (1/0) | Not applicable |
| Khachfe 2019 *(80)* | 11 | APC (for the whole period) | (1/0) | No |
| Meyer 2010 *(81)* | 33 | APC (for various periods) | (2/1) | No |
| Brenner 2017 *(83)* | 42 | APC (for various periods) | (1-4/0-3) | Yes ^(3,4,6,9)^ |
| Brenner 2019 *(84)* | 45 | APC (for various periods) | (4-5/3-4) | Yes ^(3,4,6,9)^ |
| Fedewa 2019 *(85)* | 16 | APC (for the whole period) | (1/0) | No |
| Melnitchouk 2018 *(86)* | 16 | APC (for various periods) and AAPC^3^ | (1-2/0-1) | Yes ^(6)^ |
| Siegel 2019 *(88)* | 10 | AAPC^1^ | One time segment | Not applicable |
| Al-Zalabani 2020 *(89)* | 21 | APC (for various periods) | (3/2) | Yes ^(6)^ |
| Augustus 2018 *(90)* | 15 | APC (for various periods) | (1-3/0-2) | No |
| Domati 2014 *(92)* | 23 | APC (for the whole period) | (1/0) | Yes ^(6,9)^ |
| Vuik 2019 *(94)* | 27 | APC (for various periods) | (1-2/0-1) | Yes ^(9)^ |
| Shafqat 2015 *(95)* | 12 | APC (for the whole period) | (1/0) | No |
| Siegel 2017 *(96)* | 14 | APC (for various periods) and AAPC^4^ | (1-3/0-2) | Yes ^(4)^ |
| Rahman 2015 *(98)* | 18 | AAPC | One time segment | Not applicable |
| Van Beck 2018 *(100)* | 40 | APC (for various periods) | (1-2/0-1) | Yes ^(9)^ |
| Wong 2020 *(101)* | 10 | APC (for various periods) and AAPC^2^ | (1-2/0-1) | Not applicable |
| Russo 2019 *(104)* | 17 | APC (for various periods) | (1-2/0-1) | Yes ^(4,9)^ |
| Perdue 2014 *(107)* | 20 | APC (for various periods) | (1-2/0-1) | Yes ^(4,6)^ |
| Siegel 2020 *(110)* | 22 | APC (for various periods) and AAPC^3^ | (2-5/1-4) | No |
| Patel 2016 *(112)* | 42 | APC (for various periods) and AAPC^3^ | (2-4/1-3) | Yes ^(3,6)^ |
| Vardanjani 2018 *(113)* | 10 | APC (for the whole period) | (1/0) | No |
| Siegel; Fedewa 2017 *(114)* | 40 | APC (for various periods) | (2-5/1-4) | Yes ^(6,9)^ |
| Siegel 2012 *(117)* | 17 | APC (for various periods) | (2-3/1-2) | Yes ^(4,6)^ |
| Siegel; Medhanie 2019 *(118)* | 10 | AAPC | One time segment | Yes ^(6)^ |
| Sung 2019 *(119)* | 16/20 | APC (for various periods) and AAPC^2^ | (1-3/0-2) | Yes ^(6,9)^ |
| Rafiemanesh 2016 *(120)* | 6 | APC (for the whole period) | (1/0) | Yes ^(9)^ |
| Reggiani-Bonetti 2013 *(124)* | 23 | APC (for various periods) | (2/1) | Yes ^(6)^ |
| Phipps 2012 *(126)* | 10 | AAPC | One time segment | Yes ^(5,6)^ |
| Innos 2018 *(129)* | 10 | APC (for various periods) and AAPC^4^ | (1-3/0-2) | No |
| Siegel 2014 *(130)* | 10 | APC (for various periods) and AAPC^3^ | (1-2/0-1) | Yes ^(4)^ |
| Sia 2014 *(131)* | 11 | AAPC | One time segment | No |
| Rejali 2018 *(132)* | 12 | APC (for various periods) and AAPC^4^ | (3/2) | Yes ^(6)^ |
| Sarakarn 2017 *(133)* | 24 | APC (for various periods) and AAPC^3^ | (2/1) | Yes ^(3,6)^ |
| Keum 2014 *(134)* | 35 | Not reported | Not reported | No |
| Singh 2014 *(135)* | 22 | Biannual percentage change (for the whole period) | (1/0) | Not applicable |
| Sun 2020 *(137)* | 55 | APC (for various periods) and AAPC^4^ | (1-5/0-4) | Yes ^(4,6,7,9)^ |
| Troeung 2017 *(141)* | 26 | APC (for the whole period) | (1/0) | Yes ^(6)^ |
| Wen 2018 *(148)* | 16 | Average Biannual percentage change (for the whole period) | (1/0) | Yes ^(3,4)^ |
| Wu 2018 *(150)* | 39 | APC (for various periods) and AAPC^4^ | (1-3/0-2) | Yes ^(4,6)^ |
| Zhang 2018 *(155)* | 30 | APC (for various periods) and AAPC^4^ | (1-2/0-1) | Yes ^(6,9)^ |
| Zhou 2015 *(156)* | 5/7 | APC (during two predefined periods) | (1/0) | No |
| Zorzi 2019 *(158)* | 12 | APC (for various periods) | (1-3/0-2) | Yes ^(6,9)^ |

**Abbreviations:** APC: Annual percentage change, AAPC: Average annual percentage change.

**Estimated measure/ Definition of APC and AAPC:**

1. Study stated a clear explanation of AAPC calculation
2. Study explicitly stated the difference in calculation between APC and AAPC
3. Study stated the difference between APC and AAPC by reporting the number of years covered for each measure
4. Study did not state the difference between APC and AAPC

**Parameters setting:**

1. Study specified the used modeling method (Grid search or Hudson’s).
2. Study reported the chosen minimum APC difference worth detecting.
3. Study reported the minimum number of joinpoints selected.
4. Study reported the maximum number of joinpoints selected.
5. Study reported the AAPC segment ranges that were selected.
6. Study reported the chosen model selection method.
7. Study reported the method used for estimating confidence intervals.
8. Study reported the chosen autocorrelated errors option.
9. Study reported selecting a linear or log-linear model.
